# AFM-Fold: Rapid reconstruction of protein conformations from AFM images

**DOI:** 10.1016/j.bpj.2026.06.022

**Published:** 2026-06-19

**Authors:** Tsuyoshi Kawai, Yasuhiro Matsunaga

**Affiliations:** 1 RIKEN Center for Computational Science, 7-1-26 Minatojima-minami-machi, Chuo-ku, Kobe, Hyogo 650-0047, Japan; 2 Graduate School of Science and Engineering, Saitama University, 255 Shimo-Okubo, Sakura-ku, Saitama-shi, Saitama 338-8570, Japan

## Abstract

High-speed atomic force microscopy (HS-AFM) enables direct visualization of protein dynamics under near-physiological conditions, yet its intrinsic limitation to surface topography prevents atomic-level structural characterization. We present AFM-Fold, a generative AI-based framework that reconstructs three-dimensional protein conformations directly from AFM images. AFM-Fold combines a group-equivariant convolutional neural network, which extracts low-dimensional collective variables (CVs) from AFM images, with a guided diffusion process that generates conformations consistent with the inferred CVs. Using pseudo-AFM images of adenylate kinase, AFM-Fold accurately reproduced not only the open and closed conformations, but also a continuous range of intermediate conformations spanning the open-closed transition. Application to 159 experimental HS-AFM frames of the flagellar protein FlhA_C_ further demonstrated that AFM-Fold yields conformations more consistent with experimental images than rigid-body fitting of the crystal structure, and it captures time-correlated domain motions that reflect underlying conformational dynamics. AFM-Fold enables rapid, physically plausible structure estimation from individual AFM images, typically within 1 min per frame, without relying on molecular dynamics simulations. This unified and computationally efficient pipeline opens a route to high-throughput structural analysis of HS-AFM movies.

## Significance

High-speed atomic force microscopy (HS-AFM) enables real-time observation of biomolecular dynamics under near-physiological conditions, yet extracting three-dimensional atomic structures from surface topography images remains challenging. Existing approaches face a trade-off: rigid-body fitting is fast but cannot capture conformational flexibility, while molecular dynamics-based flexible fitting captures structural changes but requires hours to days of computation per frame. AFM-Fold overcomes this trade-off by combining a symmetry-aware neural network with guided diffusion-based structure generation, reconstructing physically plausible conformations in approximately 1 min per frame without molecular dynamics simulations. This speed enables high-throughput structural analysis of entire HS-AFM movies of hundreds of frames through AFM-conditioned generative reconstruction, yielding detailed structural insight into time-correlated domain motions that reflect underlying conformational dynamics.

## Introduction

High-speed atomic force microscopy (HS-AFM) enables direct visualization of biomolecular dynamics in solution under near-physiological conditions, providing unprecedented insights into the relationship between biomolecular conformational dynamics and biological function.^1^^,^^2^ However, AFM measurements are inherently limited to surface topography, and the achievable spatial resolution is fundamentally constrained by the finite size of the cantilever tip.^3^^,^^4^ To address these limitations, there is a compelling need for computational modeling approaches that can integrate experimental AFM data with atomic-resolution three-dimensional structural models.

One approach is rigid-body fitting, in which template structures are selected from structural databases or molecular dynamics (MD) simulations and then exhaustively rotated and translated to find the best pose matching the AFM image.^5^^,^^6^^,^^7^^,^^8^^,^^9^^,^^10^^,^^11^^,^^12^^,^^13^ Although powerful, this approach cannot account for conformational flexibility or changes; consequently, rigid-body fitting often fails to provide accurate structural models when the template library lacks conformations that match the AFM image. In real biological systems, the function of biomolecules is often coupled with conformational flexibility or dynamics. Thus, methods accounting for such conformational flexibility are required for analyzing and interpreting experimental AFM data.

In recent years, methods have been proposed that utilize MD or Monte Carlo (MC) simulation to generate conformations consistent with AFM images. For example, in flexible fitting,^14^^,^^15^ the agreement with an AFM image is defined as an additional potential energy function *V*_AFM_, and MD simulations are driven to minimize it. In flexible fitting, sampling is often trapped in local minima, making optimization slow when separated by high barriers. If the global minimum is desired, prohibitively long simulations must be performed to ensure adequate sampling of the conformational space. Normal-mode-based approaches achieve substantially faster per-frame processing on standard hardware. NMFF-AFM^16^^,^^17^ employs an iterative scheme in which the linear normal-mode response is consecutively applied to gradually changing conformations, enabling the reconstruction of large-amplitude anharmonic transitions. AFMFit^18^ takes a different strategy, combining FFT-based pose search with a nonlinear normal-mode method to avoid structural distortions inherent in linear extrapolation and to efficiently process hundreds of molecular images into a conformational ensemble. However, these approaches rely on MD force fields or elastic-network-derived normal-mode bases, so the accessible conformational space is shaped by the choice of model parameterization and the number of modes retained. Another approach is to use a 3D Gaussian mixture model combined with MC sampling to estimate 3D low-resolution protein structures with conformational flexibility.^19^^,^^20^

In the field of AI-based structure modeling or prediction, sequence-to-structure models such as AlphaFold 2,^21^^,^^22^^,^^23^ AlphaFold 3,^24^ and Boltz-1^25^ have demonstrated unprecedented precision in predicting the 3D fold of a protein. These models can be regarded as generative AI models that generate atomistic conformational ensembles from a given sequence. In principle, these generative AI models can generate not only specific peak structures present in the training data, but also diverse structural conformations. Moreover, it is known that the generation process can be navigated by applying conditioning or external forces during the generation stage. Recently, guiding the generative process of diffusion-based structure prediction models, including AlphaFold 3 and Boltz-1, has emerged as an approach aimed at generating targeted conformational ensembles. For example, in the context of NMR and X-ray crystallography data analysis,^26^ it has been demonstrated that imposing guided restraints during the generative process enables the generation of ensembles consistent with experimental observations. Similarly, for cryo-EM data,^27^ the incorporation of global or local density-map fitting as restraints has been proposed as a means of navigating the predicted conformations toward agreement with the experimental data.

In this study, we introduce AFM-Fold, which extends these ideas to AFM data analysis by employing structural restraints extracted from AFM images to guide the sampling trajectories of pre-trained protein structure generative models. Specifically, in the first step, a group-equivariant convolutional neural network (CNN)^28^ estimates low-dimensional CVs (such as inter-domain distances) from AFM images, and then these estimated values are used as restraints to guide the generative process, enabling the rapid sampling of 3D conformations consistent with AFM images. We validated AFM-Fold in twin experiments. First, using adenylate kinase (AK, PDB: 1AKE^29^ and 4AKE),^30^ we estimated the underlying CV values and accurately reconstructed conformations close to the ground truth. Second, we applied AFM-Fold to real experimental HS-AFM data from the flagellar protein FlhA_C_ (PDB: 3A5I)^31^ and confirmed that it yields conformations more consistent with AFM images than the crystal structure fitted by rigid-body fitting calculations. Because the correlation coefficient—the standard metric for AFM fitting—is dominated by global molecular pose and is insensitive to subtle conformational differences, we further validate the estimates using an independently trained embedding model that provides a structurally meaningful metric space. Furthermore, we confirmed that AFM-Fold captures the time-correlated domain motions that reflect underlying conformational dynamics.

A distinctive feature of our framework, AFM-Fold, is that all steps—from training data generation, model training, and inference to structure generation—are integrated within a single unified pipeline, without relying on time-consuming processes such as MD simulations. This enables rapid reconstruction of structures from AFM images while reducing computational cost, allowing the observation of structural dynamics from hundreds of consecutive HS-AFM images. AFM-Fold provides a framework for observing conformational transitions and functional mechanisms underlying HS-AFM images.

## Materials and methods

**Figure 1:**
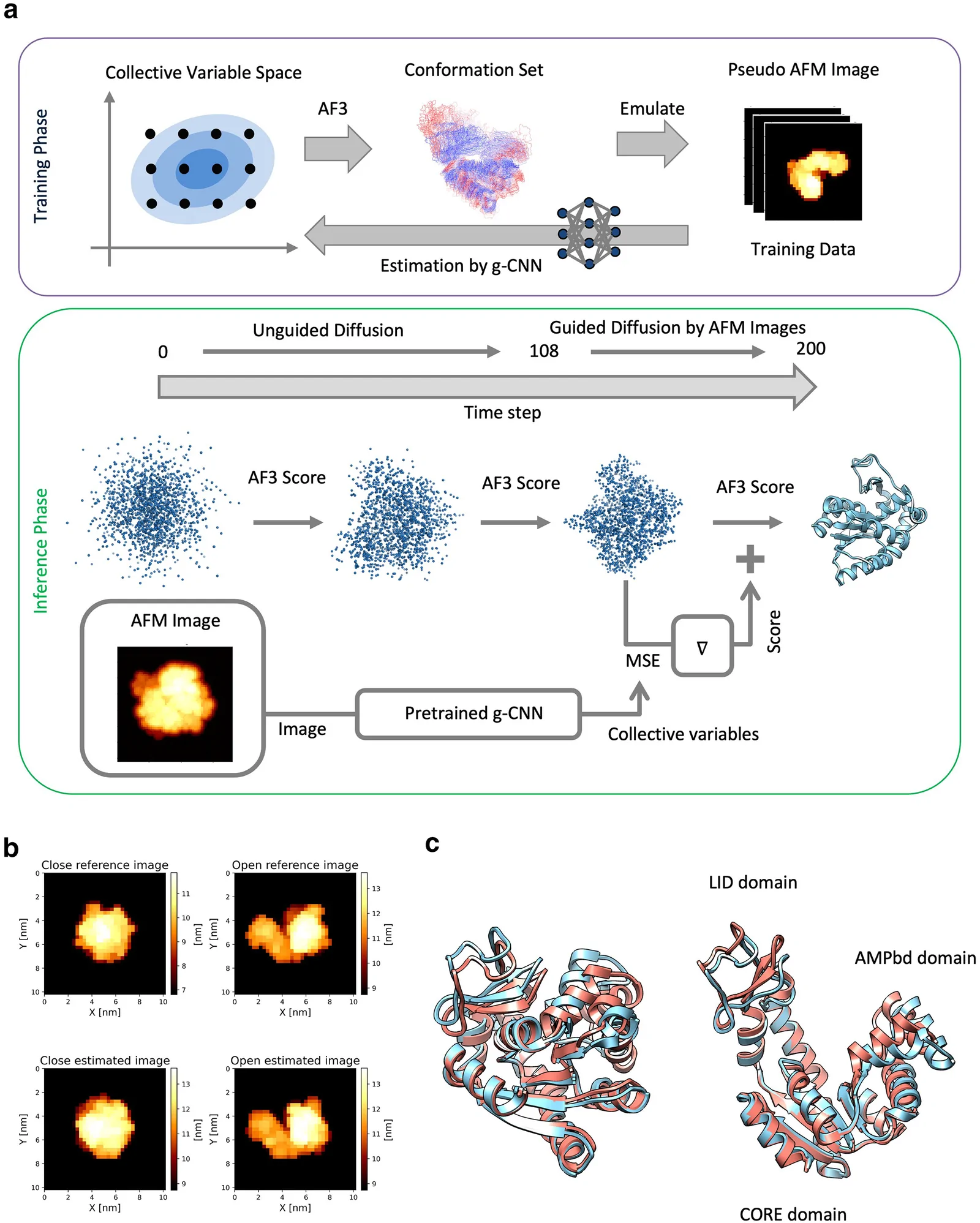
Schematic overview of AFM-Fold and outcome. (a) The AFM-Fold framework consists of two phases. Training phase (top): target CV values are placed on a grid in the CV space and used to navigate AlphaFold 3, generating a diverse set of conformations. Pseudo-AFM images are then rendered from each conformation under uniformly sampled 3D orientations, and the resulting (image, CV) pairs serve as the training data for the g-CNN. Inference phase (bottom): the pre-trained g-CNN estimates CV values from an input AFM image; during the generative process of AlphaFold 3, where the diffusion step i progresses from 0 to 200, the MSE between the estimated CVs and those of the generated conformation is computed when i > 108, and its gradient is added to the AlphaFold 3 score to guide the generation toward a conformation consistent with the AFM image. (b and c) Results of structure estimation from pseudo-AFM images of AK. In (b), the upper panels show the reference pseudo-AFM images generated from the crystal structures (closed: 1AKE, open: 4AKE). The lower panels show the pseudo-AFM images generated from the structures estimated by AFM-Fold. In (c), the blue structures represent the reference conformations, and the red structures are the corresponding AFM-Fold estimates.

Our AFM-Fold framework leverages AlphaFold 3 (here, we used the open-sourced Protenix model,^32^ a PyTorch reimplementation of AlphaFold 3) to estimate the underlying 3D conformation from a given AFM image. To generate 3D structures consistent with AFM images using AlphaFold 3, we follow two main steps: (1) first, we estimate low-dimensional CVs for the target molecule from the given AFM image using a pre-trained CNN. For CVs, we use inter-domain distances, assuming multi-domain proteins as a typical case. (2) Next, we use the estimated CVs to add restraints to AlphaFold 3 during structure generation, producing 3D structures that are consistent with the AFM image (see Figure 1).

In this section, we first present the overall computational protocol of AFM-Fold in AFM-fold computational protocol, providing a step-by-step overview of the complete pipeline. We then describe the two key components in detail: the group-equivariant CNN used to estimate CVs from AFM images (group-equivariant CNNs) and the method for navigating the diffusion process of AlphaFold 3 using the estimated CVs (navigating AlphaFold 3 with inter-domain distance restraints). Finally, in related work, we provide an overview of related work.

### AFM-Fold computational protocol

Our AFM-Fold framework consists of two main steps: (1) estimation of CVs from AFM images using a group-equivariant CNN (g-CNN; group-equivariant CNNs) and (2) navigating the generative process of AlphaFold 3 using the estimated CVs (navigating AlphaFold 3 with inter-domain distance restraints). However, additional steps are still required for preparing training data for the g-CNN, training, inference, and evaluation. In this subsection, we describe these steps.

#### Step 1: Preparation of training data

To train the g-CNN to learn the relationship between AFM images and underlying 3D structures, we construct training data by generating pseudo-AFM images from 3D conformations and pairing them with ground-truth CV labels. In this study, these conformations are generated by applying diverse CV-based restraints to AlphaFold 3, which is far cheaper than MD simulations and allows us to densely sample intermediate conformations so that the g-CNN can learn to interpolate across the conformational space. Specifically, we broadly covered the CV space by specifying the CV values on a grid. First, we obtain a reference structure *X*_ref_ with AlphaFold 3 with no restraints. Then, target CV vectors $\left\{\phi_{\text{perturb}}^{\;i}\right\}$ are placed on a grid around *ϕ*(*X*_ref_) such that, for each dimension *i*,

(1) $$\phi_{\text{perturb},d}^{i}\in \left[\min\left(0.0\;nm,\phi^{i}\left(X_{\text{ref}}\right)-5.0\;nm\right),\phi^{i}\left(X_{\text{ref}}\right)+5.0\;nm\right].$$

The grid used a step of 0.5 nm for each axis. Then, the generation with AlphaFold 3 is conducted using each $\left\{\phi_{\text{perturb}}^{\;i}\right\}$ as the restraints for the navigating process. For both AK and FlhA_C_, we used the inter-domain distances (distances between the centers of mass of C*α* atoms in the domains) as the CVs. To exclude conformations with geometric violations (e.g., steric clashes), we performed geometric sanitization based on MolProbity scores (Table S2). The conformations that passed this sanitization were used as the training set for the g-CNN; 112 conformations remained for AK and 64 for FlhA_C_.

From each conformation in the training set, pseudo-AFM images were generated by simulating the scanning of a virtual probe tip over the molecular surface and recording the maximum height at each pixel, following the morphology-based approach of our previous work^4^; the specific parameter settings are summarized in Table S3. Each molecular structure was uniformly rotated over all 3D orientations and randomly translated within the image area, placed on the stage (*z* = 0), and scanned by the simulated tip. In total, 5,000,000 pseudo-AFM images were generated by randomly sampling conformations and poses, of which 80% were used for training and 20% for validation. For AK, we used a smaller tip radius and finer resolution than typical experiments to validate the method with simulation-generated ground truth; the results thus represent an idealized upper bound. For FlhA_C_, the precise tip geometry used in the experiment is unknown; we estimated the approximate order of the tip radius using the method of Matsunaga et al.^4^ and sampled the probe radius *r* uniformly within the estimated range. Rather than optimizing all imaging parameters (stage–molecule distance, noise, etc.), we set the stage-molecule distance to zero, included only random noise, and compensated for the remaining discrepancies via histogram matching (skimage.exposure.match_histograms). This step mitigates the distribution shift between simulated and experimental images—a known challenge when training machine learning models on synthetic data—by aligning the height distributions without requiring explicit modeling of all experimental factors.

Prior to analysis, the experimental AFM images were preprocessed as follows: (1) the mean height value was subtracted from each frame to remove the baseline offset; (2) tilt correction was performed by fitting and subtracting a second-order polynomial surface; (3) each frame was scaled using RobustScaler to normalize intensity values while suppressing the influence of outliers; and (4) individual molecules were extracted from each frame via blob detection, and their trajectories across consecutive frames were obtained by frame-to-frame tracking, yielding time-series image sequences of single molecules.

#### Step 2: Training

Here, the training of the g-CNN can be regarded as supervised regression, where the task is to estimate the CV values from a given AFM image. Note that, because the CVs and the training conformational ensemble are specific to each target protein, the g-CNN must be trained separately for each protein system of interest. As the most straightforward loss function for supervised regression, we adopted the mean-squared error (MSE) loss in the CV space:

(2) $$L_{\text{CNN}}={Mean}_{i}\|\phi_{\text{est}}\left(I_{i}\right)-\phi_{\text{true},i}{\|}^{2},$$

where *I*_*i*_ denotes the *i*-th input pseudo-AFM image, *ϕ*_est_(*I*_*i*_) is the CV values estimated by the g-CNN, and *ϕ*_true,*i*_ is the corresponding ground-truth CV values. As the g-CNN architecture, we implemented a $\left(R^{2},+\right)⋊C_{8}$-equivariant CNN whose group-pooled output is invariant to in-plane rotations and translations (see Algorithm S1 for the implementation details).

All models in this paper were trained for 20 epochs with a batch size of 32, using the Adam optimizer with a learning rate of 10^−4^, on a single node equipped with an NVIDIA RTX A6000 GPU (48 GB memory). The training took approximately 2–3 days for both AK and FlhA_C_.

#### Step 3: Inference

The inference step consists of the estimation of CVs (inter-domain distances) from the AFM image by the g-CNN and the navigation of the generative process of AlphaFold 3 using the estimated CVs. In this study, we require that the MSE of the g-CNN for the generated structures be at the sub-angstrom level, which is an important resolution for distinguishing different conformations for both AK and FlhA_C_.

The computation time for the inference step was extremely short compared with flexible fitting calculations using MD simulations. Indeed, inference time was less than 1 min per image for both AK and FlhA_C_.

#### Step 4: Evaluation

To evaluate the accuracy of the estimated conformation, for AK, we computed the root-mean-square deviation (RMSD) between the estimated and the ground-truth conformations. Furthermore, since we don’t know the ground-truth conformation for FlhA_C_, we evaluated how well the estimated conformation reproduces the given AFM image using two different metrics: the correlation coefficient (c.c.) and a neural network-based embedding distance. In both cases, a simulated AFM image is rendered from the candidate conformation, and the imaging parameters (3D rotation, translation, and tip radius) are optimized by a common grid search procedure. The two metrics differ only in the objective function: for the c.c.-based evaluation, the imaging parameters are optimized to *maximize* the c.c. between the simulated and experimental images, whereas for the embedding-based evaluation, they are optimized to *minimize* the geodesic distance between the corresponding embedding vectors.

Traditionally, the similarity between an AFM image and a conformation is evaluated by rendering a simulated AFM image from the conformation and computing the c.c. against the experimental image. The imaging parameters—3D rotation, translation, and tip radius—are varied to maximize the c.c.^14^:

(3) $$c.c.\left(R\right)=\frac{\underset{p\in \text{pixels}}{\sum }H_{p}^{\left(\text{exp}\right)}H_{p}^{\left(\text{sim}\right)}\left(R\right)}{{\left[\underset{p\in \text{pixels}}{\sum }{\left(H_{p}^{\left(\text{exp}\right)}\right)}^{2}\right]}^{1/2}{\left[\underset{p\in \text{pixels}}{\sum }{\left(H_{p}^{\left(\text{sim}\right)}\left(R\right)\right)}^{2}\right]}^{1/2}}$$

where $H_{p}^{\left(\text{exp}\right)}$ and $H_{p}^{\left(\text{sim}\right)}\left(R\right)$ denote the heights of the experimental and simulated image pixels, respectively. However, the c.c. is largely dominated by the global translation and rotation, so that subtle differences in internal molecular structure manifest only as numerically small changes in the score. Moreover, the absolute value of c.c. depends on conditions such as the noise level, the number of pixels, the tip size, the molecule size, and conformation of the molecule; consequently, a given numerical improvement in c.c. cannot be directly translated into a quantitative statement about how much the underlying molecular shape has improved. While an increase in c.c. qualitatively indicates a better structural description, the lack of a universal mapping between c.c. differences and structural accuracy makes the metric difficult to interpret in practice.

To address this limitation, we trained a separate ResNet-18 model^33^ to learn an embedding space in which AFM images of structurally similar conformations are mapped to nearby points, while images of dissimilar conformations are mapped far apart, as in Dingeldein et al.^34^ This was achieved through contrastive learning,^35^ a framework in which pairs of images are labeled as “similar” or “dissimilar,” and the network is trained to bring similar pairs closer together in the embedding space. In standard contrastive learning, this similarity is defined as a binary label; however, conformational similarity is inherently continuous: two structures may differ by varying degrees. We therefore replaced the binary label with the squared difference of the inter-domain distances as a continuous structural label: image pairs from conformations with similar inter-domain distances are pulled together more strongly, while pairs from conformations with very different distances are pushed apart proportionally. To further bridge the distribution gap between simulated and experimental images (sim-to-real gap), we incorporated domain adaptation^36^ so that the network focuses on molecular shape rather than domain-specific artifacts such as noise characteristics. The resulting embeddings are approximately invariant to in-plane rotation (Figure S4) and encode structurally meaningful features that correlate strongly with inter-domain distances (see Algorithm S2 for the network architecture).

Using this trained network, we evaluated the similarity between a candidate conformation and an experimental AFM image as follows. First, a simulated AFM image was rendered from the conformation. The imaging parameters (3D rotation, translation, and tip radius) were then optimized, using the same grid search settings as for the c.c.-based fitting, to minimize the geodesic distance between the embedding vectors of the simulated and experimental images. The resulting geodesic distance *d*_geo_ = arccos(cos *θ*) on the unit hypersphere was used as the similarity metric: a smaller distance indicates a better match between the conformation and the experimental observation.

More specifically, the contrastive loss is a soft Gaussian kernel-weighted variant of the NT-Xent loss,^35^ in which positive-pair weights decay smoothly with the L2 distance between structural labels (*σ* = 1.0, temperature *τ* = 0.1). Domain adaptation was implemented via a domain-adversarial neural network framework^36^ with sigmoid alpha scheduling (*γ* = 10) that gradually strengthens the adversarial signal during training, complemented by a maximum mean discrepancy loss (*λ*_mmd_ = 0.5). Training used the same set of simulated AFM images as for the g-CNN (5,000,000 images), combined with experimental images in a 50:50 batch mixture (batch size 256), with the experimental data cyclically resampled to compensate for their smaller number. One epoch is defined as a full pass over all training images; the model was trained for 100 epochs with the Adam optimizer (learning rate 10^−4^, gradient clipping at max norm 1.0) on a single GPU.

### Group-equivariant CNNs

In AFM image analysis, similar to single-particle analysis in cryo-EM, the orientation of the molecule on the surface is unknown. Determining the correspondence between a 2D image and the underlying 3D structure therefore requires an exhaustive search over possible poses, which is computationally expensive. Furthermore, any misalignment directly degrades the accuracy of the structural estimate. In cryo-EM, this issue is mitigated by averaging over a large number of micrographs, but in HS-AFM, where we aim to estimate the structure from each individual frame to capture conformational dynamics, even small alignment errors are unacceptable.

Before describing our approach, we first discuss a more conventional alternative that we chose not to adopt. In rigid-body fitting, a candidate 3D structure is placed in a trial orientation, a simulated image is rendered from it, and the pixel-wise similarity (e.g., c.c.) between this rendered image and the observed AFM image is computed; the pose and structure are then iteratively updated to maximize this similarity. One could apply the same strategy within the generation process of AlphaFold 3, scoring each intermediate structure against the reference AFM image at every step. While this pixel-level comparison could in principle yield more detailed image reconstructions than our CV-based approach, we empirically found that it tends to trap structures in local minima. This happens because the image similarity landscape is highly multimodal and extremely sensitive to small translational and rotational shifts; under a feasible amount of alignment search, the optimization receives inaccurate gradients that mislead the generation process.

To avoid these problems, we need a comparison measure between AFM images and 3D structures that is robust to in-plane rotations and translations, and that can be evaluated efficiently across many AFM frames. Our strategy is to use a *group-equivariant* CNN (g-CNN)^28^^,^^37^^,^^38^^,^^39^ that estimates low-dimensional, structure-related CVs—such as distances between protein domains—directly from each AFM image, bypassing the need for explicit alignment altogether.

A standard CNN is equivariant to translations (shifting the input shifts the output in the same way), but not to rotations. A g-CNN extends this to rotations by modifying feature indexing and kernel weight sharing so that the network becomes equivariant under a chosen discrete rotation group.^28^^,^^37^ Because the rotational symmetry is built into the architecture rather than learned from data, the g-CNN achieves high accuracy with far less training data and shorter training time than a conventional CNN would require. In the final layers, group pooling and spatial pooling collapse the equivariant features into a vector that is *invariant* to both rotations and translations, so the same molecular conformation produces the same CV estimate regardless of how it is oriented or positioned in the AFM image. We use this g-CNN to estimate CVs from each single AFM image, which then guide the structure generation in AlphaFold 3 (as described in navigating AlphaFold 3 with inter-domain distance restraints). The g-CNN is trained in a supervised manner on pairs of simulated pseudo-AFM images and their known CV values, computed from the corresponding 3D conformations.

### Navigating AlphaFold 3 with inter-domain distance restraints

Next, we describe a method for imposing a restraint on the generative process of AlphaFold 3 so that the CVs approach user-specified target values. Specifically, while AlphaFold 3 employs an elucidated diffusion model (EDM) formulation,^40^ we compute the likelihood of the generated structure with respect to the specified CVs and add it to the original AlphaFold 3 score as a driving force that increases this likelihood. With this modified score, the generated structures become explicitly conditioned on the CVs.

Let *a* denote an amino acid sequence and *X* = (*x*_1_, …, *x*_*m*_) the 3D coordinates of all atoms, where $x_{i}\in R^{3}$. We write $X_{t_{i}}$ for the state at diffusion time *t*_*i*_(*T* > *t*_1_ > *t*_2_ > … > *t*_*N*_ > 0). Sampling the generative process of AlphaFold 3 can be written as the stochastic EDM sampler:

(4) $$\begin{array}{cc} & X_{t_{i+1}}=X_{t_{i}}-\eta \left(t_{i+1}-{\hat{t}}_{i}\right)\nabla_{X_{t_{i}}}\log p_{t_{i}}\left(X_{t_{i}}|a\right)+\lambda {\left({\hat{t}}_{i}^{2}-t_{i}^{2}\right)}^{1/2}\epsilon ,\\ & \nabla_{X_{t_{i}}}\log p_{t_{i}}\left(X_{t_{i}}|a\right)\approx -\left(X_{t_{i}}-\text{Denoiser}\left(X_{t_{i}}|a\right)\right)/{\hat{t}}_{i}. \end{array}$$

Here, ${\hat{t}}_{i}$ denotes a time larger than *t*_*i*_, defined as ${\hat{t}}_{i}=\left(1+\gamma_{i}\right)t_{i}$, with a parameter *γ*_*i*_ that is set to 0.8 when *t*_*i*+1_ > 1.0, and 0 otherwise. The coefficients *η* and *λ* are hyperparameters called the *noise scale* and *step scale*, respectively, and are set to *η* = 1.5 and *λ* = 1.003. Denoiser(⋅|*a*) represents a denoiser conditioned by coevolutionary information about *a*.

Let $\phi_{\text{target}}\in R^{D}$ denote the target CV coordinate, where *D* is the dimension of the CVs. By Bayes’ rule, the conditional score decomposes as

(5) $$\nabla_{X_{t}}\log p_{t}\left(X_{t}\left|\phi_{\text{target}},a\right)\right)=\nabla_{X_{t}}\log p_{t}\left(X_{t}|a\right)+\nabla_{X_{t}}\log p_{t}\left(\phi_{\text{target}}|X_{t},a\right).$$

Therefore, we can bias the stochastic sampler by adding the guidance term:

(6) $$X_{t_{i+1}}=X_{t_{i}}-\eta \left(t_{i+1}-{\hat{t}}_{i}\right)\left(\nabla_{X_{t_{i}}}\log p_{t_{i}}\left(X_{t_{i}}|a\right)+\nabla_{X_{t_{i}}}\log p_{t_{i}}\left(\phi_{\text{target}}|X_{t_{i}},a\right)\right)+\lambda {\left({\hat{t}}_{i}^{2}-t_{i}^{2}\right)}^{1/2}\epsilon$$

so that the process is guided toward $\phi \left(X_{t_{N}}\right)$ ≈ *ϕ*_target_, where $\phi :R^{3m}\to R^{D}$ is a differentiable mapping from structures to this feature space.

In this work, we replace the guidance score by the gradient of a squared-error loss:

(7) $$L_{\text{MSE}}\left(X\right)={\left\|\phi \left(X\right)-\phi_{\text{target}}\right\|}_{2}^{2},$$

(8) $$\nabla_{X_{t}}\log p_{t}\left(\phi_{\text{target}}|X_{t},a\right)\approx -\eta_{t}\nabla_{X_{t}}L_{\text{MSE}}\left(X_{t}\right),$$

which is consistent with an isotropic Gaussian model $p_{t}(\phi_{\text{target}}|X_{t},a)$ ∝ $\exp(-\|\phi \left(X_{t}\right)-\phi_{\text{target}}{\|}_{2}^{2}/\left(2\sigma_{t}^{2}\right))$, in which case $\eta_{t}=1/\left(2\sigma_{t}^{2}\right)$. The optimal selection of *η*_*t*_ is analyzed in Section S1, and a concise summary of all key hyperparameters governing the guided diffusion process is provided in Table S1.

### Molecular dynamics simulation

To verify the AFM-Fold framework, we performed MD simulations of AK and FlhA_C_. For AK, we performed an all-atom MD simulation to compare the distribution of conformational states sampled by MD with those generated by an AlphaFold 3 clone (Protenix^32^) for training the g-CNN. Simulations were conducted using the Amber ff14SB force field^41^ with the TIP3P-FB water model and ions,^42^ starting from the open crystal structure (PDB: 4AKE). The simulations were carried out at 300 K and 1 atm using OpenMM,^43^ and the trajectory was extended to 450 ns to ensure sufficient sampling of the conformational space.

For FlhA_C_, due to computational time constraints, we did not conduct all-atom MD simulations. Instead, we generated conformational ensembles using a set of MD surrogate models: AlphaFlow^44^ and BioEmu,^45^ as well as AlphaFold 2 with multiple sequence alignment (MSA) subsampling^46^ using ColabFold.^23^ These generative approaches enabled us to obtain a diverse ensemble of conformations, facilitating a comprehensive comparison with the distribution generated by our AFM-Fold framework.

### Related work

Generative AI-based approaches employing diffusion models or flow models enable us to efficiently sample molecular conformations in a multiscale manner. For example, recent structure-generation foundation models, including AlphaFold 3 and Boltz-1, employ diffusion-based formulations. In particular, Boltz-2^47^ introduced *boltz-steering*, a framework that imposes a customized potential to navigate the sampling toward physically plausible conformations. These models largely follow a “one sequence–one structure” paradigm, which does not focus on producing diverse conformational ensembles or capturing conformational flexibility.

Several approaches have been proposed to address this problem. For example, the MSA subsampling technique^46^ and its extensions^48^^,^^49^^,^^50^ increase the diversity of MSAs by masking parts of the MSA. Another powerful direction is the development of ensemble generative or MD surrogate models. An early contribution in this area is the Boltzmann generator,^51^ which was designed to learn and reproduce the Boltzmann distribution of small, specific peptides. More recently, models have been developed to generate diverse structural ensembles from sequences by learning from extensive MD simulation data across a wide range of proteins.^44^^,^^45^^,^^52^^,^^53^^,^^54^^,^^55^^,^^56^ In these methods, obtaining sufficiently long MD trajectories for training requires substantial computational resources, and such models inevitably inherit the force-field biases present in the training data.

To mitigate this issue, recent studies have explored conditioning ensemble generative models on experimental measurements or physical prior knowledge. Examples include methods that generate structural ensembles conditioned on experimental observables such as X-ray crystallographic electron densities, nuclear Overhauser effect,^26^ structural constraints derived from experimental data,^57^ and cryo-EM-consistent modeling.^27^^,^^58^ Following this direction, AFM-Fold enables AFM-conditioned conformation generation, guiding AlphaFold 3’s prior distribution toward modeling more dynamic conformational states captured in HS-AFM images.

## Results and Discussion

The estimation pipeline of AFM-Fold operates as follows: given an input AFM image, the trained g-CNN directly estimates the CV values (inter-domain distances) from the image; these estimated CV values are then used as restraints to navigate the generative process of AlphaFold 3, which produces a 3D conformation consistent with the input AFM image. Because the group-pooled output of the g-CNN is invariant to in-plane rotations and translations, the same conformation yields the same CV estimate regardless of how the molecule is oriented or positioned in the image, eliminating the need for explicit image alignment.

### Verification using pseudo-AFM images: Adenylate kinase

AK is a well-studied monomeric enzyme known for its large-scale conformational transitions. It is composed of three relatively rigid domains: the central CORE domain (residues 1–29, 68–117, and 161–214), the AMP-binding domain (AMPbd; residues 30–67), and the lid-like ATP-binding domain (LID; residues 118–167). Experimental and computational studies have suggested that upon ligand binding, the enzyme undergoes a transition from an inactive open conformation to an active closed conformation (see Figure 1c). Here, we evaluated how accurately AFM-Fold reconstructs 3D conformations of AK from artificially created pseudo “experimental” AFM images.

#### Training data generation and g-CNN accuracy

**Figure 2:**
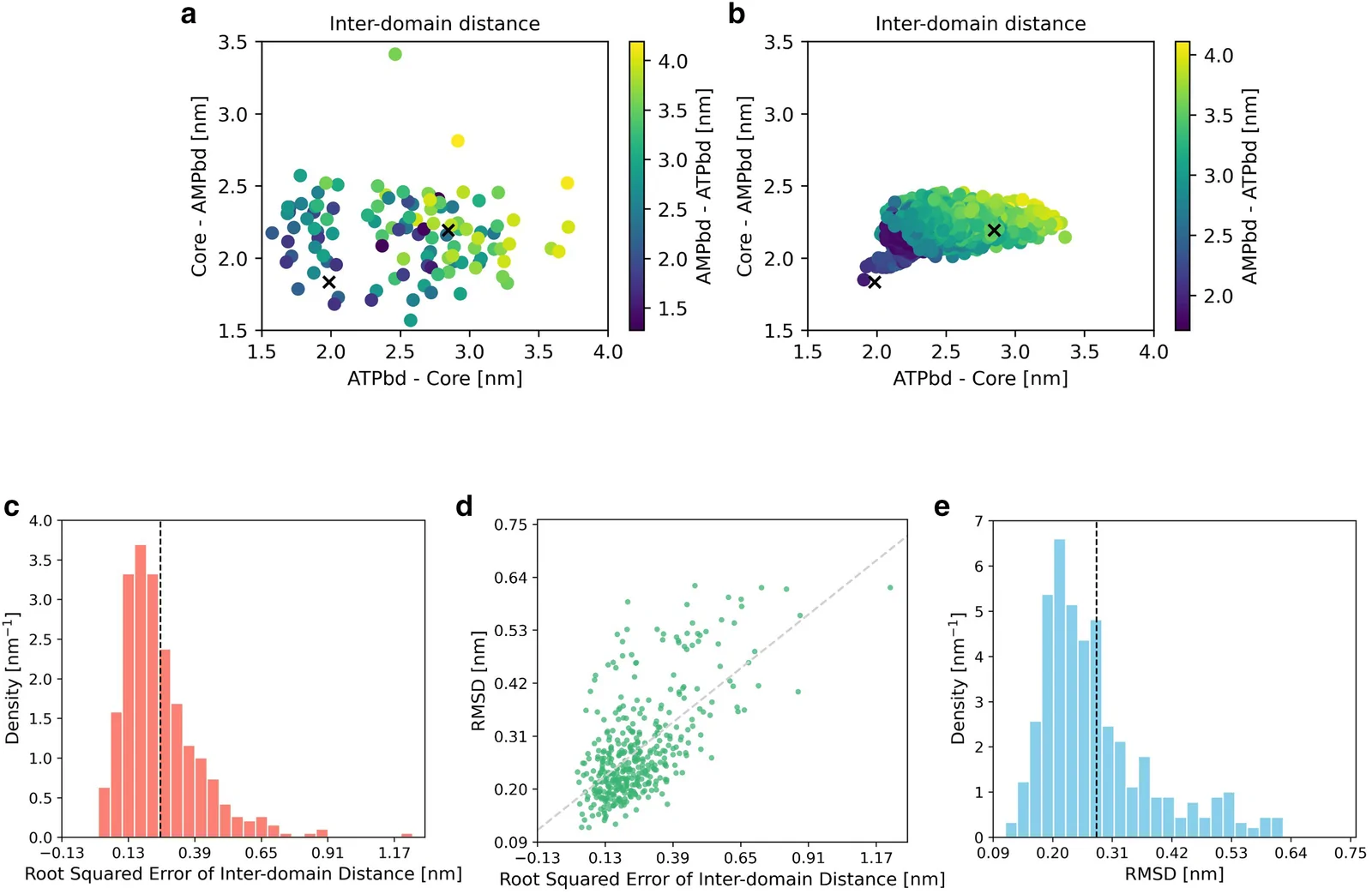
Training data and estimation accuracy of AFM-Fold. (a) Projection of training data into the inter-domain distance space. The two cross marks represent the open (4AKE) and closed (1AKE) crystal structures from PDB, respectively. (b) Projection of a 450-ns MD simulation trajectory into the inter-domain distance space. (c) Distribution of the root-squared error in the inter-domain distance space between the g-CNN’s estimations and the ground-truth. The inter-domain distance was estimated by the trained g-CNN from pseudo-AFM images, which were generated from randomly selected molecular dynamics simulation conformations. The average of the root-squared errors is indicated by the broken line (0.254 nm). (d) Scatter plot of the root-squared error in the inter-domain distance space and the heavy-atom RMSDs from the ground-truth conformation. The heavy-atom RMSD was computed between the conformation generated with AlphaFold 3 navigating using the estimated CV values and the ground-truth conformation. (e) Density of the heavy-atom RMSDs. The average of the RMSD is indicated by the broken line (0.281 nm).

We generated a conformational ensemble by navigating AlphaFold 3 to target CV values—defined as the inter-domain distances for all domain pairs (LID–CORE, CORE–AMPbd, and AMPbd–LID)— sampled on a regular grid (see Section S1 for details of the navigation schedule). The generated ensembles are shown with their MolProbity scores in Figure S5. The resulting ensemble adequately covers the broad conformational space of AK, encompassing both the closed (PDB: 1AKE) and open (PDB: 4AKE) crystal structures (Figures 2a and 2b)). We then trained the g-CNN on this ensemble and evaluated its estimation accuracy using pseudo-AFM images generated from randomly selected MD conformations (see Table S3 for the image settings). The root-mean-squared error in the CV space is 0.254 nm (Figure 2c), apart from a few outliers exceeding 0.500 nm—sufficiently small to resolve the intermediate conformational states between the open and closed forms of AK.

#### Accuracy of conformations estimated by AFM-Fold

We first applied AFM-Fold to pseudo-AFM images of the closed and open crystal structures of AK (Figures 1b and 1c). Note that these pseudo-AFM images were generated under idealized imaging conditions (small tip radius, fine resolution, no noise; see materials and methods), so the accuracy reported here represents an upper bound on what can be achieved; the impact of more realistic conditions is assessed in the robustness analysis below. The RMSDs between the reconstructed and ground-truth conformations were 0.216 nm (closed) and 0.176 nm (open), both well below the 0.715-nm RMSD separating the two crystal structures, confirming that AFM-Fold clearly distinguishes between the two states. Accordingly, the pseudo-AFM images generated from the estimated conformations are visually similar to the input images, with c.c. values (Equation 3) of 0.997 (closed) and 0.998 (open).

We next assessed reconstruction accuracy over a broader range of conformations using pseudo-AFM images generated from randomly selected MD snapshots. Note that these MD conformations span a continuous distribution across the open-closed landscape and are not classified into discrete states; the RMSD and CV errors reported below therefore reflect accuracy over this continuum. Most RMSDs are distributed around the mean of 0.281 nm, with a small number of outliers (Figures 2d and 2e). Inspection of the outlier cases revealed two contributing factors (Figure 2d). First, depending on how the molecule is oriented on the stage (i.e., which face of the protein contacts the surface), the resulting AFM image may look similar regardless of whether the conformation is open or closed. Because the g-CNN is trained on pseudo-AFM images rendered from uniformly sampled 3D orientations, it must learn to estimate CVs from all possible views; however, certain out-of-plane orientations produce nearly degenerate images for structurally distinct conformations, making it inherently difficult for the g-CNN to estimate the correct inter-domain distances from such views. Second, even when the estimated CV values are accurate, the reconstructed RMSD can still be elevated because the CV-structure mapping is not unique: distinct domain arrangements—such as hinge-bending versus shear-like motions—can yield similar inter-domain distances while producing structurally different conformations. Indeed, Figure 2d shows that several data points with small CV errors nonetheless exhibit elevated RMSDs, confirming that the CV-structure degeneracy—rather than CV estimation inaccuracy—is the dominant source of reconstruction error in these cases.

#### Robustness against noise and imaging conditions

To evaluate the applicability of AFM-Fold to real experimental data, we investigated its robustness against two key factors that degrade image quality: measurement noise and imaging conditions (image resolution and probe tip radius).

**Figure 3:**
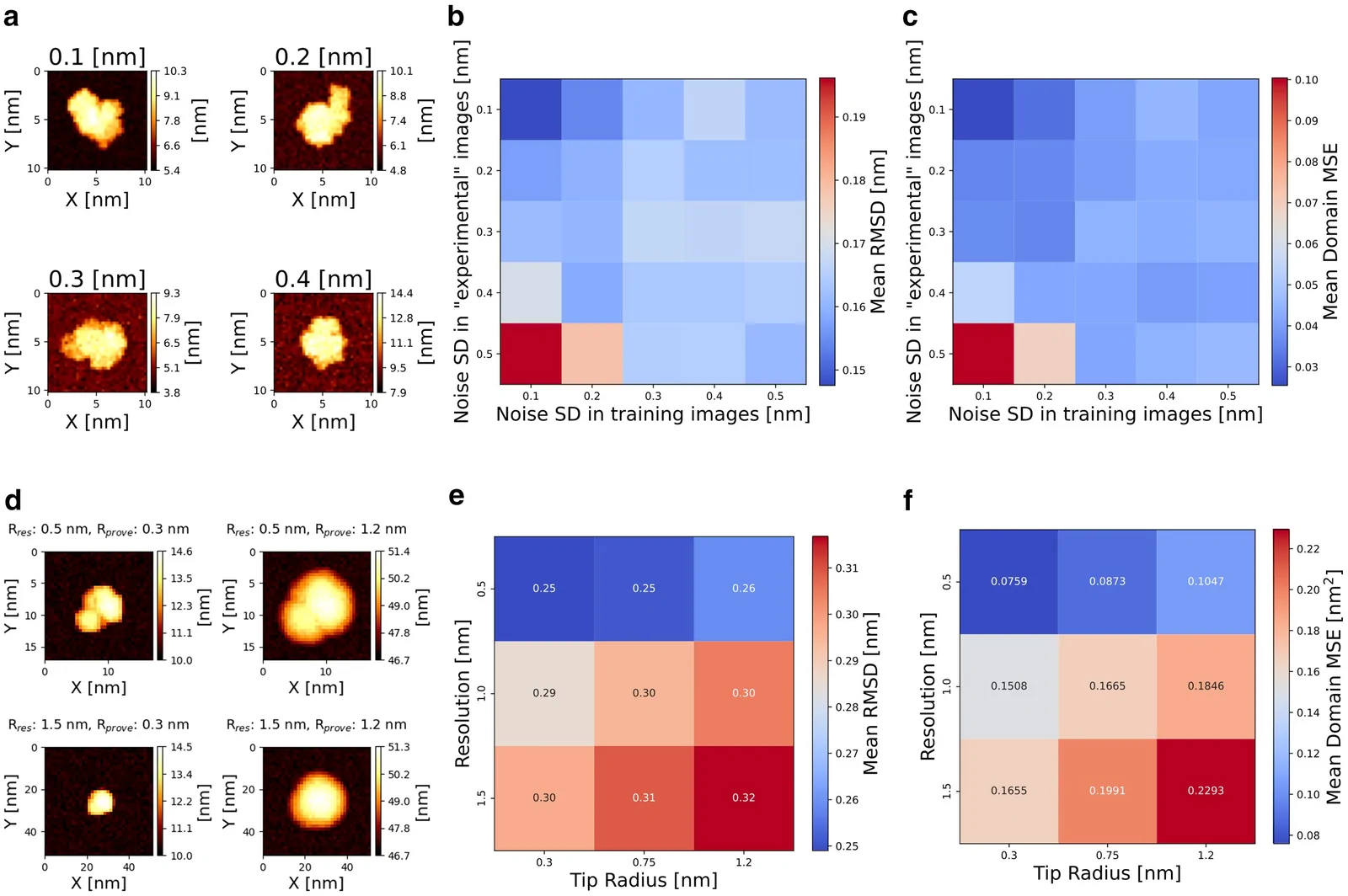
Robustness against noise and imaging conditions. (a) Examples of pseudo-AFM images with Gaussian noise at various standard deviations (0.1–0.4 nm), generated from randomly selected MD conformations. (b) Mean RMSD between the estimated and ground-truth structures as a function of noise levels in both the training and “experimental” pseudo-AFM images. Each value was computed from 20 independent estimations. (c) Mean-squared error (MSE) of the inter-domain distances under the same noise conditions as in (b). (d) Pseudo-AFM images generated with different combinations of the image resolution Rres and the probe tip radius Rprobe. (e) Mean RMSD between the estimated and ground-truth structures as a function of the tip radius and the image resolution. (f) MSE of the inter-domain distances as a function of the tip radius and the image resolution.

We first examined the effect of noise by systematically varying the Gaussian noise levels added to both the training and “experimental” pseudo-AFM images (Figure 3a). The resulting heat maps of the mean RMSD (Figure 3b) and the inter-domain distance MSE (Figure 3c) reveal two distinct trends. Adding noise to the “experimental” images consistently led to substantial reductions in estimation accuracy, whereas adding noise to the training data alone had a comparatively minor effect; indeed, the best performance was achieved with noise-free training data. These results indicate that AFM-Fold is sensitive to noise in the input images but does not require noisy training data to handle noisy inputs.

We next assessed the influence of imaging conditions by varying the image resolution *R*_res_ and the probe tip radius *R*_probe_ (Figure 3d). The heat maps of the mean RMSD (Figure 3e) and the inter-domain distance MSE (Figure 3f) show that image resolution is the dominant factor governing estimation accuracy. Because AK is a relatively small molecule, coarse resolution causes it to appear as a nearly featureless round spot, making it difficult for the g-CNN to distinguish different conformations; accordingly, degrading the resolution leads to a dramatic increase in estimation error. In contrast, increasing the probe tip radius has a comparatively modest effect on accuracy, indicating that AFM-Fold is relatively robust to tip broadening effects.

#### Correlation coefficient as an evaluation metric

When applying AFM-Fold to real experimental data, ground-truth structures are unavailable and RMSD cannot be computed. We therefore examined whether the c.c. between pseudo-AFM images and the reference images can serve as a reliable proxy for structural accuracy.

**Figure 4:**
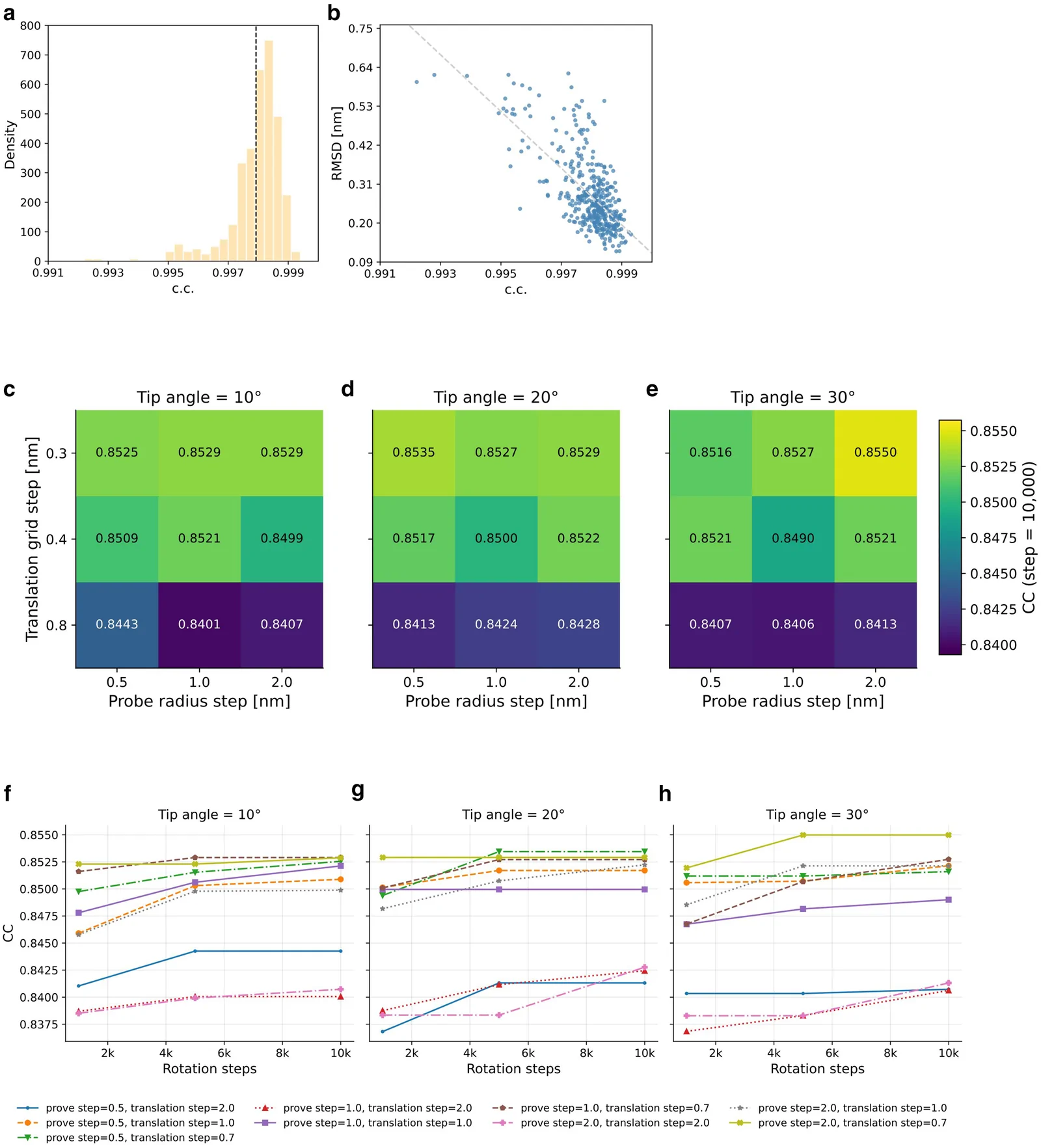
Variability of the c.c. and its dependence on fitting parameters. (a) Distribution of c.c. values between the “experimental” pseudo-AFM images and pseudo-AFM images generated from the estimated conformations after rigid-body fitting. The dashed line indicates the mean value (0.998). (b) Scatter plot of the c.c. versus the RMSD from the ground-truth conformation. The dashed line shows the linear fit. (c–e) Maximum c.c. achieved for different combinations of the translation grid step and the probe radius step at tip angles of 10° (c), 20° (d), and 30° (e). (f–h) Maximum c.c. as a function of the number of rotation search steps at tip angles of 10° (f), 20° (g), and 30° (h). Different curves correspond to different combinations of the probe radius step and translation grid step.

The c.c. values are concentrated in a narrow range of 0.995–0.999, with a mean of 0.998 (Figure 4a). Despite this narrow distribution, a clear negative correlation exists between the c.c. and the RMSD (Figure 4b), confirming that the c.c. is indeed a meaningful indicator of structural agreement. Notably, however, even a modest difference of ∼0.01 in c.c. can correspond to a wide range of RMSD values (Figure 4b), implying that small improvements in c.c. often reflect substantial differences in structural accuracy. This observation underscores the importance of precise c.c. evaluation: the metric is reliable, but the structurally relevant signal resides in very small numerical differences. This inherent compression of structural information into numerically small differences motivates the introduction of a complementary embedding-based metric, described below, in which conformational differences are encoded as interpretable spatial distances.

We next investigated how sensitively the c.c. depends on the parameters of the rigid-body fitting procedure. Using a single experimental AFM image and the crystal structure of FlhA_C_ (discussed in the next section) as the target, we performed repeated fitting trials in which the structure was randomly rotated and then optimized over translation and probe tip radius at various grid step sizes (Figures 4c–4e). The c.c. was highly sensitive to the translation grid step: a coarse translation search frequently failed to locate the optimal c.c. value. In contrast, the c.c. was relatively insensitive to the probe radius grid step. For the rotational search, the c.c. converged in most cases by approximately 5,000 rotation steps (Figures 4f–4h). Based on these results, we adopted a translation step of 0.7 nm, a probe radius step of 1 nm (2 nm–6 nm for FlhA_C_), a tip angle of 20°, and 10,000 rotation steps for all subsequent analyses, which we consider sufficient for reliable c.c. evaluation. With these settings, the rigid-body fitting takes approximately 3 min per structure on a single GPU.

### Application to real experimental AFM images: A flagellar protein FlhA_C_

**Figure 5:**
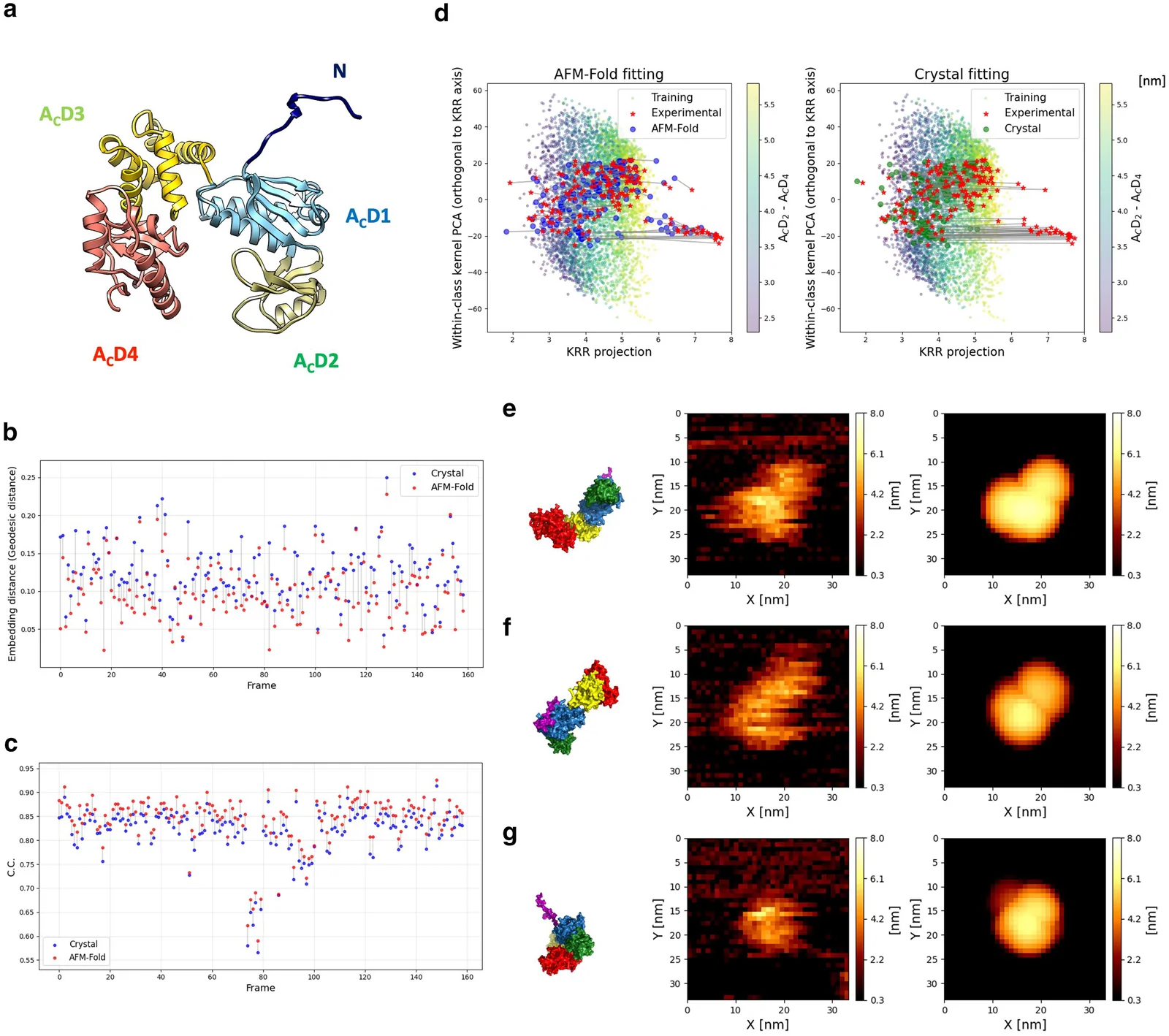
Structural feature space analysis of FlhA_C_ using ResNet-18 embeddings. (a) Structure of FlhA_C_ with domain coloring. (b) Per-frame geodesic distance in the embedding space (AFM-Fold versus crystal). (c) Per-frame c.c. (AFM-Fold versus crystal). (d) Two-dimensional projection of the embedding vectors. The *x* axis corresponds to the A_C_D_2_–A_C_D_4_ inter-domain distance (*r* = 0.898); the *y* axis captures the leading orthogonal variation (explained variance 35.9%). Colored points: training data colored by the A_C_D_2_–A_C_D_4_ distance. Left: AFM-Fold estimates (blue) and experimental images (red stars). Right: crystal structure (green) and experimental images (red stars). (e–g) Three representative cases in which the AFM-Fold estimate differs visibly from the crystal structure (e and f: more open; g: more closed). For each case: (left) the AFM-Fold-estimated 3D structure (domain-colored as in a); (center) the experimental AFM image; (right) the pseudo-AFM image rendered from the AFM-Fold structure after rigid-body fitting that maximizes the c.c. The c.c. value (between the experimental image and the rendered pseudo-AFM image) is shown above each row.

FlhA is a key component of the flagellar protein export system, and its C-terminal domain, FlhA_C_, helps transport proteins by assisting their binding to the exporter. Structurally, it comprises four domains (A_C_D_1_, A_C_D_2_, A_C_D_3_, and A_C_D_4_) and a linker to the transmembrane domain (FlhA_TM_) (Figure 5a). Previous studies using X-ray crystallography and mutational analysis indicate that hinge motions among these domains influence transport activity and motility.^31^

Here, to evaluate the applicability of AFM-Fold to real AFM data, we used HS-AFM images of FlhA_C_ acquired by Inoue et al.^59^ The images were recorded with a spatial resolution of approximately 1.0 nm/pixel (59 × 59nm^2^ field of view, 60 × 60 pixels) and a frame interval of 66 ms, with the sample immobilized on mica in a buffer containing 20 mM NaCl and 10 mM Tris-HCl (pH 8.0). We estimated the conformations of FlhA_C_ using AFM-Fold from 159 HS-AFM images of the FlhA_C_ monomer. It should be noted that, whereas conventional flexible fitting methods for capturing structural changes typically require hours to days of computation per AFM image even with a coarse-grained model,^14^ AFM-Fold allows estimation of conformations from each AFM image in approximately 1 min on a single GPU once the g-CNN has been trained (see conclusion for a discussion of the training cost). This made it practical to process all 159 AFM images in this study without a per-frame MD simulation.

#### Training of the g-CNN

Conformations for training the g-CNN were generated by navigating AlphaFold 3 with CV values on a grid as described in step 2: training. As the CVs, we used the inter-domain distances of domain pairs, A_C_D_1_–A_C_D_4_, A_C_D_2_–A_C_D_3_, and A_C_D_2_–A_C_D_4_. The generated ensembles are shown with their MolProbity scores in Figure S6. The g-CNN was trained on the training data to learn the relationship between AFM images and the CVs.

#### Correlation and embedding-based evaluation of estimated conformations

We applied AFM-Fold to all 159 experimental AFM images. For each frame, we rendered simulated AFM images from the AFM-Fold estimate (and, separately, from the crystal structure) and performed two types of rigid-body fitting over the imaging parameters (3D rotation, translation, and tip radius): one maximizing the c.c. and the other minimizing the geodesic distance in the embedding space (see step 4: evaluation). Both fittings used the same grid search settings whose convergence was validated for the c.c. metric (Figures 4c–4h); although a dedicated convergence study was not conducted for the embedding-based objective, the shared parameter space and search procedure make comparable convergence expected, which was also confirmed empirically by inspecting individual fitting trajectories.

The per-frame c.c. showed consistent improvement for AFM-Fold over the crystal structure (Figure 5c), confirming that the AFM-Fold-estimated conformations better reproduce the experimental AFM images than the static crystal structure. However, the improvement margins are modest. This reflects an intrinsic degeneracy in AFM imaging: distinct 3D conformations can yield similar AFM images due to the convolution effects of the probe tip, finite resolution, and measurement noise. Under realistic imaging conditions, even the closed crystal structure can produce pseudo-AFM images of comparable similarity to those of open conformations. Moreover, the c.c. is dominated by the overall molecular envelope and is inherently insensitive to fine internal structural rearrangements, as demonstrated for AK (Figure 4a); consequently, even meaningful conformational differences manifest as only small numerical changes in c.c. values. This fundamental insensitivity of the c.c. to conformational detail necessitates a complementary metric that directly encodes structural similarity.

To overcome this limitation, we validated the estimated conformations using a metric that directly encodes structural similarity: we trained a separate ResNet-18 model^33^ that maps AFM images into an embedding space in which structurally similar images are located nearby (see step 4: evaluation for training details). Because this model was trained independently of AFM-Fold, agreement in the embedding space constitutes an external validation of the estimated conformations. Using the embedding-based fitting described above, we projected the 256-dimensional embedding vectors onto two dimensions (Figure 5d). The *x* axis is the structural feature axis, computed by regressing the A_C_D_2_–A_C_D_4_ inter-domain distance onto the embedding vectors (correlation *r* = 0.898). The *y* axis captures the leading direction of within-class variation orthogonal to this axis (explained variance 35.9%), representing variability unrelated to the A_C_D_2_–A_C_D_4_ distance. The resulting scatterplot, colored by the A_C_D_2_–A_C_D_4_ distance, shows clear separation along the *x* axis, confirming that the embeddings successfully encode conformational features. More specifically, because the embedding vectors lie on a unit hypersphere, we used the geodesic distance *d*_geo_ = arccos(cos *θ*) rather than the Euclidean distance as the dissimilarity measure for the dimensionality reduction. The *x* axis was obtained by kernel ridge regression with a geodesic radial basis function kernel $K=\exp\left(-d_{\text{geo}}^{2}/2\sigma_{K}^{2}\right)$ (*σ*_*K*_ = 0.5, regularization *λ* = 10^−3^). The *y* axis was obtained by first removing the *x* axis component via Gram–Schmidt orthogonalization on the kernel matrix and then applying kernel PCA to the residual within-class covariance and taking the first principal component. We further verified empirical rotation invariance of the embeddings (Figure S4): for the same molecular pose rendered at 8 in-plane rotation angles, the cosine similarity was 0.978 ± 0.021, and the geodesic distance was 0.179 ± 0.113, well separated from random-pair baselines (cosine similarity 0.924 ± 0.049, geodesic distance 0.374 ± 0.125).

In this structural feature space, the AFM-Fold estimates (blue dots in Figure 5d, left) cluster more closely around the experimental images (red stars) than the crystal structure does (green dots in Figure 5d, right). This is particularly noteworthy because AFM-Fold does not use the ResNet-18 model at all—it relies solely on the g-CNN for CV estimation—yet its estimates are independently validated by the ResNet-18 embedding space. Quantitatively, the mean geodesic distance between experimental images and AFM-Fold estimates was 0.101 ± 0.044, compared with 0.125 ± 0.046 for the crystal structure (Figure 5b). This separation is substantially more interpretable than the corresponding c.c. difference, because the embedding distances are directly anchored to structural similarity by construction.

Figures 5e–5g present representative cases in which the AFM-Fold estimation differs visibly from the crystal structure: (e) and (f) adopt more open conformations, while (g) is more closed.

We note that the molecular orientations shown in these panels differ between cases (for example, the two open conformations in (e) and (f) are displayed in markedly different poses). This is a consequence of our evaluation procedure rather than a physical observation: the pose of each structure is determined by an independent rigid-body fit to its own AFM image, so different conformations naturally settle into different optimal orientations, and the quantity being compared is the similarity score at the optimum, not the orientation itself. These displayed poses should therefore not be interpreted as in-plane or out-of-plane reorientations of the molecule on the substrate. In an actual HS-AFM experiment, imaging relies on firm sample-substrate attachment that permits internal domain motions while largely preserving the overall molecular orientation, and our per-frame analysis does not aim to track such absolute orientations.

Overall, both the c.c. and the embedding-based evaluation consistently indicate that AFM-Fold captures genuine conformational features present in the experimental AFM images more accurately than the static crystal structure.

#### Conformational dynamics

**Figure 6:**
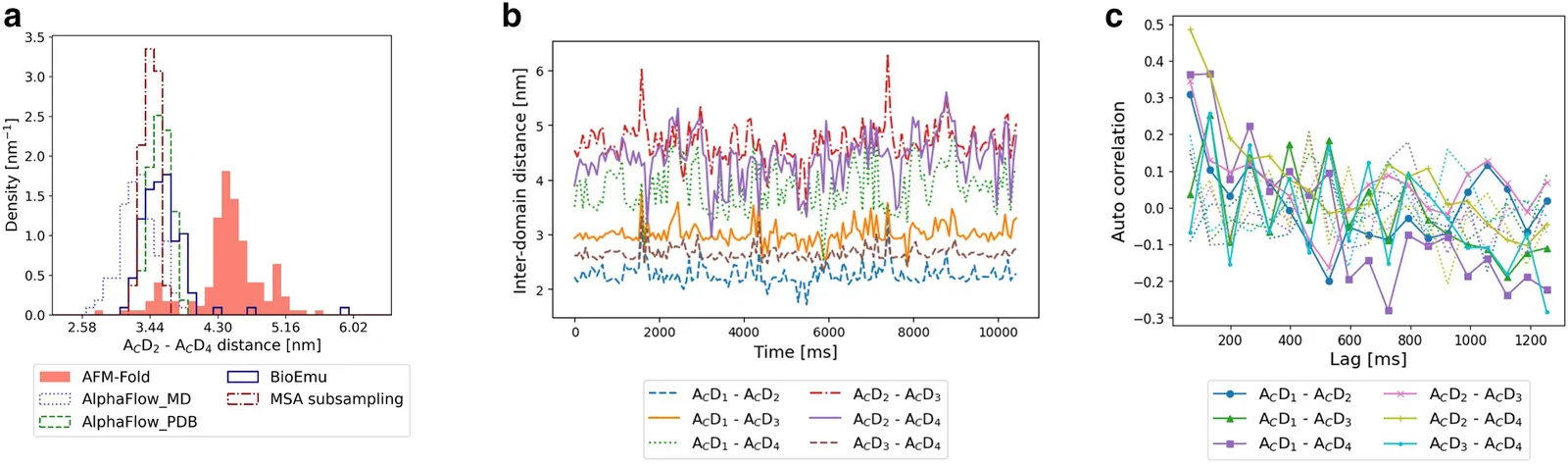
Conformational dynamics of FlhA_C_ estimated by AFM-Fold. (a) Distribution of the A_C_D_2_–A_C_D_4_ inter-domain distance estimated by AFM-Fold (red shaded) compared with AI-based structure generation models: AlphaFlow,^44^ BioEmu,^45^ and AlphaFold 2 with MSA subsampling.^46^ (b) Time series of the six inter-domain distances estimated by AFM-Fold for the consecutive 159 experimental AFM frames. (c) Auto-correlation functions of the inter-domain distances as a function of time lag. Solid lines show the results from AFM-Fold estimations, while dashed lines correspond to temporally shuffled surrogate data.

To characterize the conformational dynamics captured by AFM-Fold, we computed the distribution of the A_C_D_2_–A_C_D_4_ inter-domain distance—a key indicator distinguishing between open and closed conformations—for all 159 estimated structures (Figure 6a). For comparison, we also generated conformational ensembles using AI-based structure generation models: AlphaFlow,^44^ BioEmu,^45^ and AlphaFold 2 with MSA subsampling,^46^ which are expected to approximate long-time behaviors of FlhA_C_ that are not accessible by brute-force MD simulations. Interestingly, AFM-Fold tends to estimate more open conformational states than these AI-based models. There are three possible explanations for this tendency. First, HS-AFM captures MD with a frame interval of 66 ms, which may reflect longer-timescale motions than those sampled by AlphaFlow or BioEmu, thereby enabling the observation of more open structures. Second, interactions between the molecule and the stage during AFM imaging may stabilize conformations with larger contact surface areas, thus favoring more open structures. Third, estimation errors in AFM-Fold could lead to an overrepresentation of highly open conformations with large conformational entropy.

To assess the third possibility, we examined the time series of the estimated inter-domain distances (Figure 6b). Since the 159 AFM images are consecutive frames, we can expect temporal correlations in the estimated structures if the estimation errors are sufficiently small. Indeed, the time series exhibits clear oscillatory behavior, with the distances fluctuating between larger (open) and smaller (closed) values over time. Crucially, AFM-Fold estimates each frame independently without any temporal information, yet the estimated inter-domain distances display pronounced time correlations, implying that the estimation errors are smaller than the scale of the observed fluctuations.

This temporal correlation was quantified by the auto-correlation function (Figure 6c). The auto-correlations from AFM-Fold estimations (solid lines) show a pronounced positive correlation for lag times up to 400 ms and oscillatory behavior at longer lag times. In contrast, the auto-correlations from temporally shuffled surrogate data (dashed lines) remain low (within ±0.2) across all lag times, confirming that the observed correlations are not artifacts of the estimation procedure. Overall, these results demonstrate that AFM-Fold effectively captures conformational dynamics from the time series of HS-AFM images.

## Conclusion

In this work, we have proposed a computational protocol, AFM-Fold, to estimate the 3D conformation of biomolecules from AFM images. AFM-Fold extracts features from AFM images with a group-equivariant (symmetry-aware) CNN, reducing computational cost in two ways. First, it reduces the amount of required training data, because symmetry-aware representations learn efficiently from limited images.^28^ Second, it shortens inference time: guiding AlphaFold 3 requires computing *p*_*t*_(*ϕ*_target_|*X*_*t*_, *a*); replacing this with $L_{\text{MSE}}\left(X_{t}\right)$ keeps the computational cost per structure low. In the estimations of this study, per-frame estimation finished within 1 min on a single GPU. It has the potential to serve as a useful tool for interpreting HS-AFM movies composed of hundreds of frames in both structural and dynamic aspects.

AFM-Fold circumvents MD simulations entirely, which fundamentally changes the computational cost structure compared with existing flexible fitting approaches. In conventional flexible fitting,^14^^,^^15^ each AFM frame requires an independent MD simulation lasting hours to days, making the analysis of large image sequences prohibitive. Normal-mode-based approaches such as NMFF-AFM^16^ and AFMFit^18^ achieve even faster per-frame processing (tens of seconds per frame on standard hardware) through iterative application of normal-mode linear response, and they can capture large-amplitude anharmonic motions.^17^ However, these methods rely on normal-mode bases derived from a given force field, so the accessible conformational space is shaped by the choice of force field and the number of modes retained. In contrast, AFM-Fold leverages the coevolutionary information embedded in AlphaFold 3 to generate conformations without depending on MD force fields or normal-mode bases, offering a complementary approach.

A limitation of AFM-Fold is that it requires a priori definition of CVs as features to be estimated from the AFM image. If it is challenging to define appropriate CVs a priori, one could first perform structural sampling using methods such as AlphaFlow,^44^ BioEmu,^45^ or AlphaFold 2 with MSA subsampling^46^ and then identify meaningful CVs by applying dimensionality reduction techniques like PCA to extract important features. The AFM-Fold framework is general, and as long as the chosen CVs are differentiable with respect to atomic Cartesian coordinates and can be used for guiding AlphaFold 3, a wide variety of CV types can be incorporated. For example, although we focused on inter-domain distances as CVs in this work, other CVs, such as residue-level distance restraints, can be used to enable AFM-guided reconstruction of finer-grained motions without a significant increase in computational cost. The choice of CVs also determines the resolution at which structural degeneracy can be resolved. As demonstrated in the AK analysis (Figure 2d), inter-domain distances alone cannot distinguish domain arrangements that differ in relative orientation (e.g., hinge-bending versus shear-like motions) but yield similar center-of-mass separations. When the target protein exhibits such degenerate modes of motion, incorporating additional CVs—for example, inter-domain angles or residue-level distance restraints—would be necessary to lift the degeneracy and improve reconstruction fidelity.

Another limitation is that the g-CNN must be trained separately for each protein system, because the CVs and the training conformational ensemble are defined on a per-protein basis. This per-protein preparation is not negligible: generating pseudo-AFM images for training takes some minutes, and training the g-CNN requires 2–3 days on a single GPU with 48 GB memory. However, once trained, AFM-Fold estimates a conformation from each AFM image in approximately 1 min, enabling rapid processing of tens to hundreds of frames. More broadly, the per-protein setup of AFM-Fold currently involves several steps that require user decisions: the definition of CVs, the generation of training conformations via AlphaFold 3, and the selection of guidance scheduling parameters. Among these, the guidance scheduling was systematically evaluated in this work (see Section S1), and the results provide practical guidelines that can be reused across systems, reducing the need for exhaustive per-protein tuning. The GPU hardware requirement (48 GB memory in this study) may also present a barrier for some experimental laboratories; however, cloud-based GPU services offer an increasingly accessible alternative. These per-protein setup costs are one-time investments: once completed, the trained model can be applied to all frames of a given HS-AFM movie without further intervention.

It is also important to consider the intrinsic limitations of AFM imaging when interpreting the reconstructed structures. First, each HS-AFM frame is acquired by raster scanning over a finite time (66 ms in the FlhA_C_ data set); if the molecule undergoes conformational changes during this interval, the resulting image represents a temporal average rather than an instantaneous snapshot, which could blur the distinction between neighboring conformational states. Second, the lateral resolution is fundamentally limited by the convolution of the probe tip geometry with the molecular surface; as demonstrated in our robustness analysis (Figures 3e and 3f), coarser resolution and larger tip radii degrade conformational discrimination. For the FlhA_C_ data, the spatial resolution of ∼1.0 nm/pixel and estimated tip radius of 2–6 nm imply that sub-nanometer domain rearrangements may fall below the effective resolving power. These factors set a practical lower bound on the conformational differences that AFM-Fold can reliably detect from experimental images and should be kept in mind when interpreting the modest numerical margins observed in the c.c. comparisons—where the metric is inherently insensitive to conformational detail—and the larger, structurally grounded separation in the embedding-distance comparisons (Figures 5b and 5c).

More broadly, the reliance on simulated pseudo-AFM images for training introduces an inherent distribution mismatch with respect to real experimental data: the precise origins and statistics of experimental noise, tip-sample interactions, and imaging artifacts are difficult to reproduce exactly in simulation. In this work, we adopted histogram matching as a simple and transparent adjustment, but achieving a fully realistic sim-to-real mapping remains an open challenge. Future work could address this more systematically, for example by incorporating domain adaptation techniques that explicitly account for differences between simulated and experimental data distributions.

Another limitation of AFM-Fold is that it infers conformations from a single AFM image and currently does not quantify predictive uncertainty due to image noise or molecular orientation. Extending the framework to estimate uncertainty would make the estimates easier to interpret.^60^ Moreover, AFM image sequences constitute time-series data in which poses change only slightly between adjacent frames; incorporating temporal dependencies—for example, with TimeSformer^61^ or nonlocal networks^62^—could further improve fidelity.

Finally, in this work we navigated AlphaFold 3’s sampling only using AFM images; consequently, neither structural stability nor explicit physical priors are incorporated. In the future, conditioning the prior (such as Boltz-2’s custom potentials^47^) of an ensemble generative model should enable integrated modeling that unifies experimental measurements, physical priors, and computational inference.

## Data and code availability

The code of AFM-Fold is publicly available at GitHub: http://github.com/matsunagalab/afmfold. Notebooks, scripts, and data sets to reproduce the results of this paper are publicly available at Zenodo: https://zenodo.org/records/17714204.^63^ HS-AFM data of FlhA_C_ are available upon reasonable request to the corresponding author and with the permission of the original authors.^59^

## Acknowledgments

We appreciate Tohru Minamino (The University of Osaka) and Noriyuki Kodera (Kanazawa University) for kindly providing the experimental AFM image data used in this work. This work was supported by 10.13039/501100000646JSPS KAKENHI (grant number: 23H03412), and partly supported by 10.13039/501100001700MEXT as “Program for Promoting Researches on the Supercomputer Fugaku” (Development and application of large-scale simulation-based inferences for biomolecules JPMXP1020230119) and used computational resources of supercomputer Fugaku provided by the RIKEN Center for Computational Science (project IDs: hp230209, hp240215, and hp250233).

## Author contributions

T.K. carried out the primary research, including conceptual development, data analysis, and manuscript drafting. Y.M. designed the overall research plan, provided continuous guidance, oversaw the progress of the work, and assisted in revising and contributed to the refinement of the manuscript.

## Declaration of interests

The authors declare that they have no competing interests.

## Declaration of generative AI and AI-assisted technologies in the writing process

During the preparation of this work the authors used ChatGPT (OpenAI) and Claude (Anthropic) in order to assist with language refinement and editing. After using these services, the authors reviewed and edited the content as needed and take full responsibility for the content of the publication.

## Supplemental information

Document S1. Figures S1–S6 and Tables S1–S3

Document S2. Article plus supplemental information
