# Supplementary material for: AFM-Fold: Rapid reconstruction of protein conformations from AFM images
Source: Biophys J. 2026 Jun 19;125(15):3845–61. doi: 10.1016/j.bpj.2026.06.022 (PMC13507418; doi:10.1016/j.bpj.2026.06.022)
Supplement: Supplementary file 1 [file mmc1.pdf]

Biophysical Journal, Volume *125*

Supplemental information

AFM-Fold: Rapid reconstruction of protein conformations from AFM images

Tsuyoshi Kawai and Yasuhiro Matsunaga

S1 Navigating scheduling

We investigated how the navigating schedule η_t in **??** affects the navigating of AlphaFold 3 structure generation. The conditional generation process in AlphaFold 3 is formulated as:

$$X_{t_{i+1}} = X_{t_i} - \eta(t_{i+1} - \hat{t}_i)\big(\nabla_{X_{t_i}} \log p_{t_i}(X_{t_i}|a) - \eta_{t_i} \nabla_{X_{t_i}} \mathcal{L}_{\text{MSE}}(X_{t_i})\big) + \lambda\big(\hat{t}_i^2 - t_i^2\big)^{1/2}\epsilon, \tag{S1}$$

where i propagates from 1 to N and η_t dominates the strength of the navigation.

Prior work commonly used simple schedules––e.g., a constant value [1] or a mid-trajectory sigmoid [2]–– but, to our knowledge, there has not been a systematic quantification of how these choices affect generated structures. Here, across different navigating schedules, we evaluated the trade-off between the accuracy of following the target CV values and the physical plausibility of the generated structures, and found an optimal navigating parameter set.

Navigating family and design of the sweep. We defined navigating schedules η_t from the sigmoidal family defined in Eq. S2. Specifically, we vary two hyperparameters that determine *when* navigating turns on (τ_{start}) and *how strong* it becomes at the end (y_{max}). For the two hyperparameters, we select five candidate values each: τ_{start} is chosen at evenly spaced points in $[0.4, 0.9]$, and y_{max} is chosen at evenly spaced points in $[0.6, 1.6]$. By evaluating all combinations of these choices, we obtained $5 \times 5 = 25$ distinct schedule settings in total. For each setting we generated five independent conformations and computed the averages of evaluation metrics of them.

$$\begin{aligned}
\eta_{t_i} &= \frac{y_{\text{max}}}{2}\frac{\|\delta\|_2}{\|g\|_2}\left(1 + \frac{\tanh(\alpha(\tau_i - \beta)/2)}{\tanh(\alpha\beta/2)}\right),\\
\tau_i &= \frac{i/N - \tau_{\text{start}}}{1 - \tau_{\text{start}}},\\
\delta &= \nabla_{X_{t_i}} \log p_{t_i}(X_{t_i}), \quad g = -\nabla_{X_{t_i}} \mathcal{L}_{\text{MSE}}(X_{t_i}),\\
\alpha &= 10.0, \quad \beta = 0.5.
\end{aligned} \tag{S2}$$

Systems and CVs Here, we describe the choice of protein system and the CVs on which restraints are applied. The way restraints are imposed can in principle depend on factors such as protein size or the specific CVs chosen, but our goal is to provide a concrete reference point that can serve as a guideline when users consider how to set the navigation strength.

In these experiments, we investigated the relationship between the navigation strength and the physical plausibility of the generated structures for both AK (214 residues) and FlhA_C (346 residues). The choice of CVs used for applying the navigation was, for AK, LID–CORE, CORE–AMPbd, and AMPbd–LID, and for FlhA_C, $\text{A}_\text{C}\text{D}_1$ –$\text{A}_\text{C}\text{D}_4$ and $\text{A}_\text{C}\text{D}_2$ –$\text{A}_\text{C}\text{D}_3$. For FlhA_C, this schedule sweep deliberately used only these two CVs as a reduced subset to keep the parameter scan tractable; the final inference reported in the main text (and the full CV set listed in Table S3) additionally includes $\text{A}_\text{C}\text{D}_2$ –$\text{A}_\text{C}\text{D}_4$, for three CVs in total. Because the guidance schedule controls the overall restraint strength rather than the specific CV set, we adopted the same schedule parameters ($\tau_{\text{start}} = 0.6$, $y_{\text{max}} = 0.6$) for the three-CV final inference. As a reference, under navigation-free conditions, AlphaFold 3 predicts structures with inter-domain distances of (2.85 nm, 2.19 nm, 3.45 nm) for AK, and (3.47 nm, 4.38 nm) for FlhA_C. We conducted two types of experiments for these proteins: (i) **in-distribution targets**, which correspond to physically plausible inter-domain distances observed in MD simulations. Specifically, (2.41 nm, 2.01 nm,2.68 nm) was chosen for AK and (2.5 nm,4.5 nm) for FlhA_C. (ii) **out-of-distribution targets**, which represent physically implausible inter-domain distances not observed in MD simulations. In this case, (1.21 nm,1.51 nm,0.53 nm) was chosen for AK and (2.0 nm,3.0 nm) for FlhA_C.

Results A close examination of the MSE and MolProbity-score landscapes for each scheduling setting reveals that, for both the in-distribution and out-of-distribution targets, the two landscapes exhibit clearly distinct behaviors. Notably, in the lower-left region of the parameter space, the relationship

between the two metrics does not follow the simple rule that high MSE corresponds to low MolProbity-score (or vice versa). Instead, when the navigation is initiated early (small τ_{start}) and the maximum guidance strength is kept low (small y_{max}), both the MSE and the MolProbity-score tend to take low values. This indicates the presence of a regime in which restraints can be applied without substantially degrading the geometric quality of the generated structures. Based on these observations, in our study we adopt $\tau_{\text{start}} = 0.6$ and $y_{\text{max}} = 0.6$ that allow effective navigation without substantially degrading structural quality. All evaluated schedules are shown in Fig. S1, and the detailed results for AK and FlhA$_C$ are presented in Fig. S2 and Fig. S3, respectively.

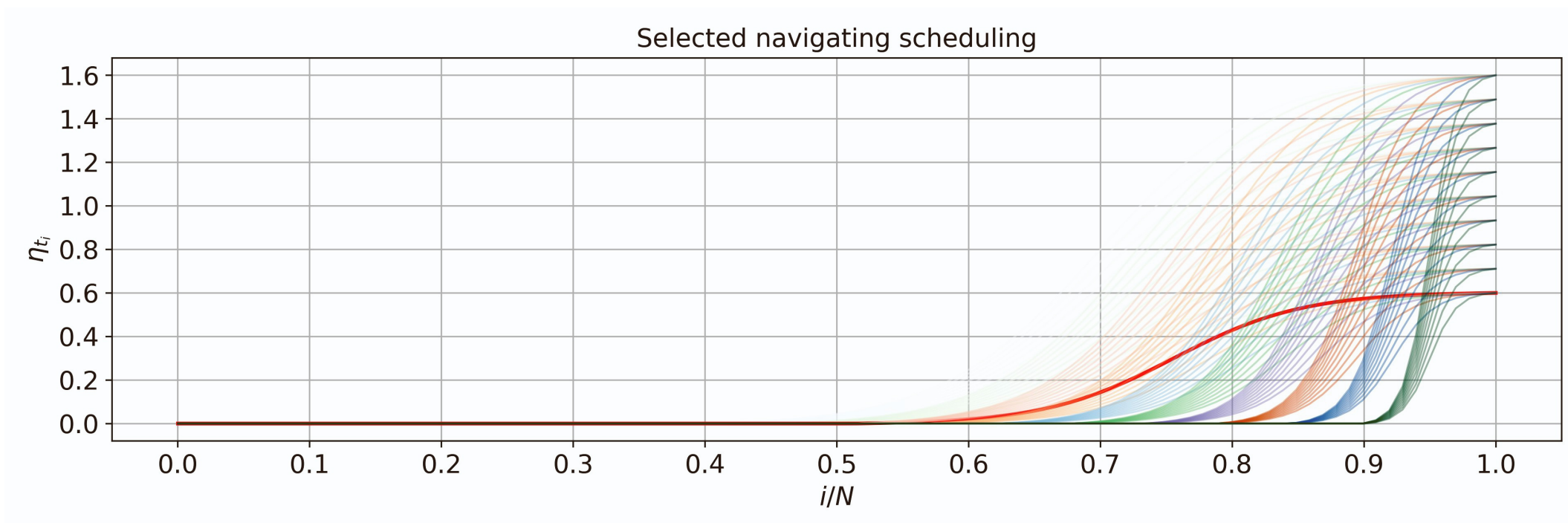

Figure S1: All navigating schedulings evaluated in this study. The setting used in our main experiments, $\tau_{\text{start}} = 0.6$ and $y_{\text{max}} = 0.6$, is highlighted with a thick red line.

S2 Model implementation details

Algorithm S1 Forward function of the $(\mathbb{R}^2, +) \rtimes C_8$-equivariant CNN

Input: $I \in \mathbb{R}^{B\times D\cdot N\times H\times W}$, $D = 1$, $N = 8$, $H = W = 35$;
channels = [3,6,6,12,12,8], kernel_size = [7,5,5,5,5,5],
$N_{\text{hidden-layers}} = 3$, $D_{\text{hidden}} = 64$, $D_{\text{out}} = N_{\text{domain-pairs}}$
Output: $\mathbf{D} \in \mathbb{R}^{B\times D_{\text{out}}}$
$I \leftarrow \text{R2Conv}(I;\ \text{in} = D\cdot N,\ \text{out} = \text{channels}[0]\cdot N,\ k = \text{kernel\_size}[0]) \rightarrow \text{InnerBatchNorm} \rightarrow \text{ReLU}$
for $i = 1 \rightarrow \text{len(channels)} - 1$ **do**
  $I \leftarrow \text{R2Conv}(I;\ \text{in} = \text{channels}[i-1]\cdot N,\ \text{out} = \text{channels}[i]\cdot N,\ k = \text{kernel\_size}[i])$
  $I \leftarrow \text{InnerBatchNorm}(I) \rightarrow \text{ReLU}(I)$
  if $i \bmod 2 = 0$ **then**
    $I \leftarrow \text{PointwiseAvgPoolAntialiased}(I; \sigma, s)$
    where $\sigma = 0.66, \quad s = \begin{cases} 2 & \text{if size odd} \\ 1 & \text{if size even} \end{cases}$
$I' \leftarrow \text{GroupPooling}(I)$ $\quad I' \in \mathbb{R}^{B\times \text{channels}[-1]\times H'\times W'}$
$I'' \leftarrow \text{PointwiseAvgPool}(I')$ $\quad I'' \in \mathbb{R}^{B\times \text{channels}[-1]\times 1\times 1}$
$\mathbf{z} \leftarrow \text{Flatten}(I'')$ $\quad \mathbf{z} \in \mathbb{R}^{B\times \text{channels}[-1]}$
$\mathbf{h} \leftarrow \text{Linear}(\mathbf{z};\ \text{in} = \text{channels}[-1],\ \text{out} = D_{\text{hidden}}) \rightarrow \text{BatchNorm1d} \rightarrow \text{ELU}$ $\quad \mathbf{h} \in \mathbb{R}^{B\times D_{\text{hidden}}}$
for $j = 1 \rightarrow N_{\text{hidden-layers}} - 1$ **do**
  $\mathbf{h} \leftarrow \text{Linear}(\mathbf{h};\ \text{in} = D_{\text{hidden}},\ \text{out} = D_{\text{hidden}}) \rightarrow \text{ReLU}(\mathbf{h})$ $\quad \mathbf{h} \in \mathbb{R}^{B\times D_{\text{hidden}}}$
$\mathbf{D} \leftarrow \text{Linear}(\mathbf{h};\ \text{in} = D_{\text{hidden}},\ \text{out} = D_{\text{out}})$ $\quad \mathbf{D} \in \mathbb{R}^{B\times D_{\text{out}}}$
return D

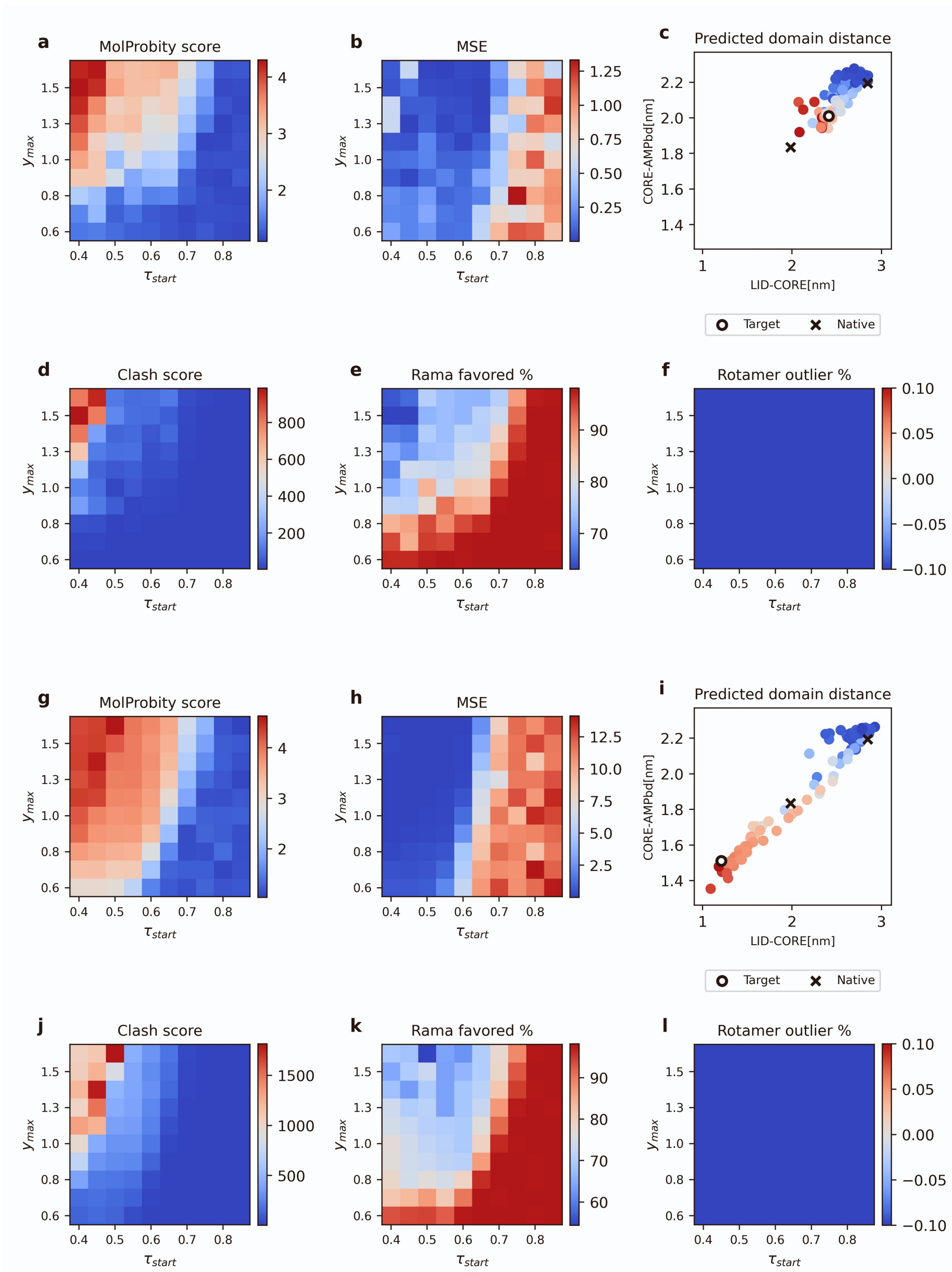

Figure S2: **Results for AK**. The upper 6 panels ((a)–(f)) shows results for the in-distribution target, and the lower 6 panels ((g)–(l)) shows results for the out-of-distribution target. **(a), (g)** Average MolProbity scores. **(b), (h)** Mean squared error (MSE). **(c), (i)** The inter-domain distance of estimated conformations (25 points, each one represents average inter-domain distance of corresponding schedule setting), with point colors representing the MolProbity scores. The cross point means that of the crystal structures (PDBID: 4AKE (open) and 1AKE (close)), and circle point means that of the target (every estimated structures were generated navigated towards this point). **(d), (e), (f), (j), (k), (l)** Evaluation metrics obtained during MolProbity scoring: clash score, Ramachandran favored rate (%), and rotamer outlier rate (%).

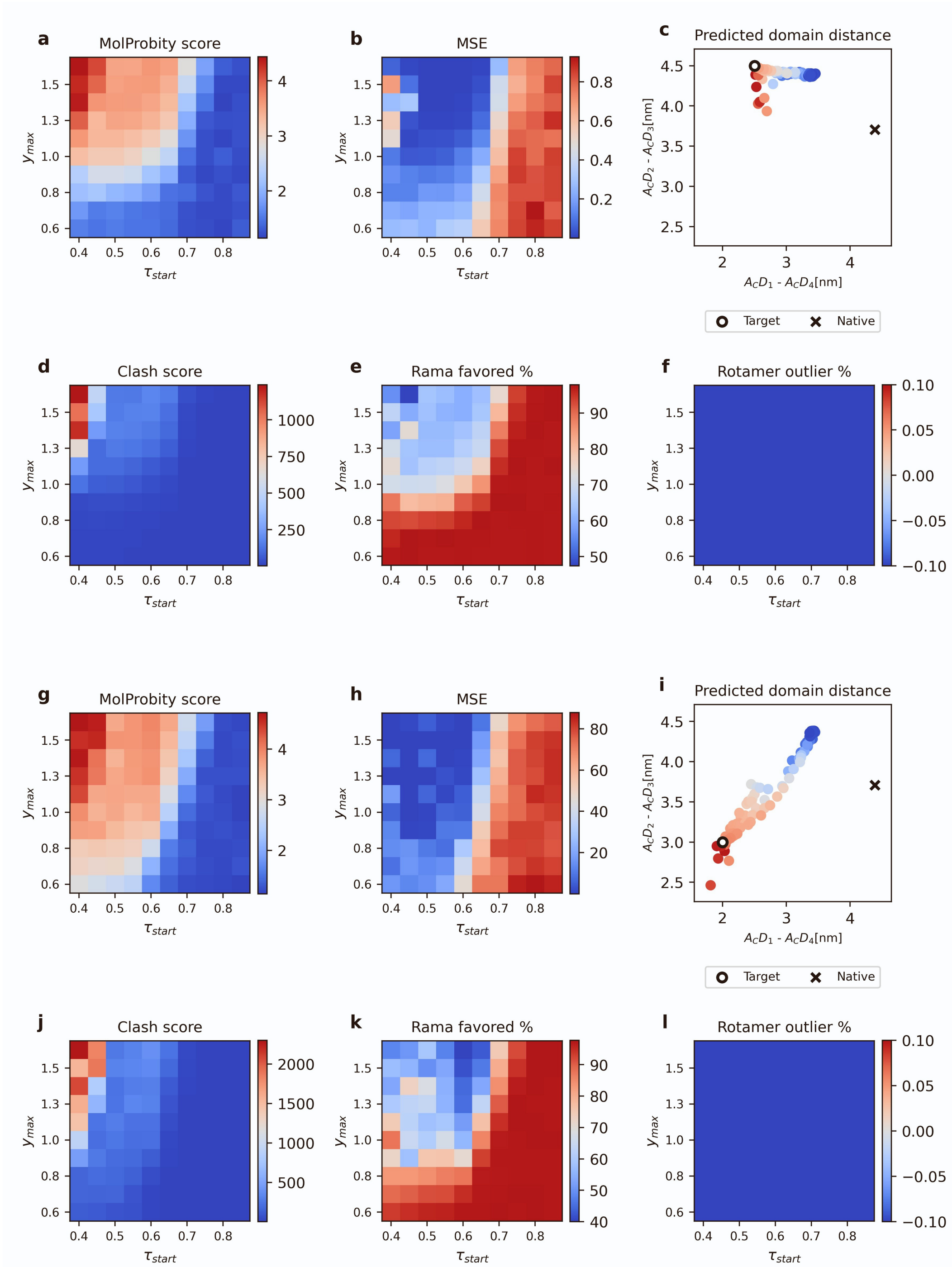

Figure S3: **Results for FlhA$_C$.** As before, the upper 6 panels ((a)–(f)) shows results for the in-distribution target, and the lower 6 panels ((g)–(l)) shows results for the out-of-distribution target. **(a), (g)** Average MolProbity scores. **(b), (h)** Mean squared error (MSE). **(c), (i)** The inter-domain distance of estimated conformations, with point colors representing the MolProbity scores. The cross point means that of the crystal structure (PDBID: 3A5I), and circle point means that of the target. **(d), (e), (f), (j), (k), (l)** Evaluation metrics obtained during MolProbity scoring: clash score, Ramachandran favored rate (%), and rotamer outlier rate (%).

Algorithm S2 Forward function of the ResNet-18 embedding model

Input: $I \in \mathbb{R}^{B\times 1\times H\times W}$, $H = W = 35$
Output: **z** $\in \mathbb{R}^{B\times 256}$ on the unit hypersphere

▷ Backbone: ResNet-18 (ImageNet pretrained, conv1 modified for 1-channel input)

$I \leftarrow$ Conv2d(I; in $= 1$, out $= 64$, $k = 7$, $s = 2$, $p = 3$) $\rightarrow$ BatchNorm2d $\rightarrow$ ReLU $\rightarrow$ MaxPool
f $\leftarrow$ ResNet-18_layers(I) ▷ **f** $\in \mathbb{R}^{B\times 512\times H'\times W'}$
h $\leftarrow$ GlobalAvgPool(**f**) ▷ **h** $\in \mathbb{R}^{B\times 512}$

▷ Projection Head

h $\leftarrow$ Linear(**h**; in $= 512$, out $= 512$) $\rightarrow$ BatchNorm1d $\rightarrow$ ReLU
h $\leftarrow$ Linear(**h**; in $= 512$, out $= 256$)

▷ L2 normalization to unit hypersphere

z $\leftarrow$ **h**$/\|$**h**$\|_2$ ▷ **z** $\in \mathbb{R}^{B\times 256}$, $\|$**z**$\|_2 = 1$
return z

S3 Supplemental tables

Table S1: Key hyperparameters for the guided diffusion process used in this study. The guidance schedule parameters were selected on the basis of the systematic sweep described in Section S1 (see also Figs. S1 to S3).

Parameter	Value	Description
Guidance schedule (Eq. S2)		
τ_{start}	0.6	Restraint onset (fraction of N)
y_{max}	0.6	Maximum guidance strength
α	10.0	Sigmoid steepness
β	0.5	Sigmoid midpoint
Training data generation		
CV grid range	± 5.0 nm around $\phi(X_{\text{ref}})$	Range of target CV values
CV grid step	0.5 nm	Spacing of target CV grid

Table S2: MolProbity score thresholds used for geometric sanitization.

Criterion	Threshold
MolProbity Score	≤ 4.0
Clash Score	≤ 70.0
Ramachandran favored (%)	≥ 90.0
Rotamer Outlier (%)	≤ 50.0

Table S3: Settings for training pseudo-AFM images.

	AK	FlhA$_C$
Lateral resolution [nm/pixel]	0.3	0.98
Pixel size [pixel × pixel]	35×35	35×35
Tip radius [nm]	$r \sim$ Uniform$(0.3, 0.6)$	$r \sim$ Uniform$(2.0, 6.0)$
Tip apex angle [degree]	$a \sim$ Uniform$(10, 30)$	$a \sim$ Uniform$(10, 30)$
Noise std. dev. [nm]	0.0	0.5
Histogram matching	—	✓
Dataset size [frames]	5M	5M
Collective variables	Inter-domain distances (LID-CORE, CORE-AMPbd, AMPbd-LID)	Inter-domain distances (A_CD_1-A_CD_4, A_CD_2-A_CD_3, A_CD_2-A_CD_4)

S4 Supplemental figures

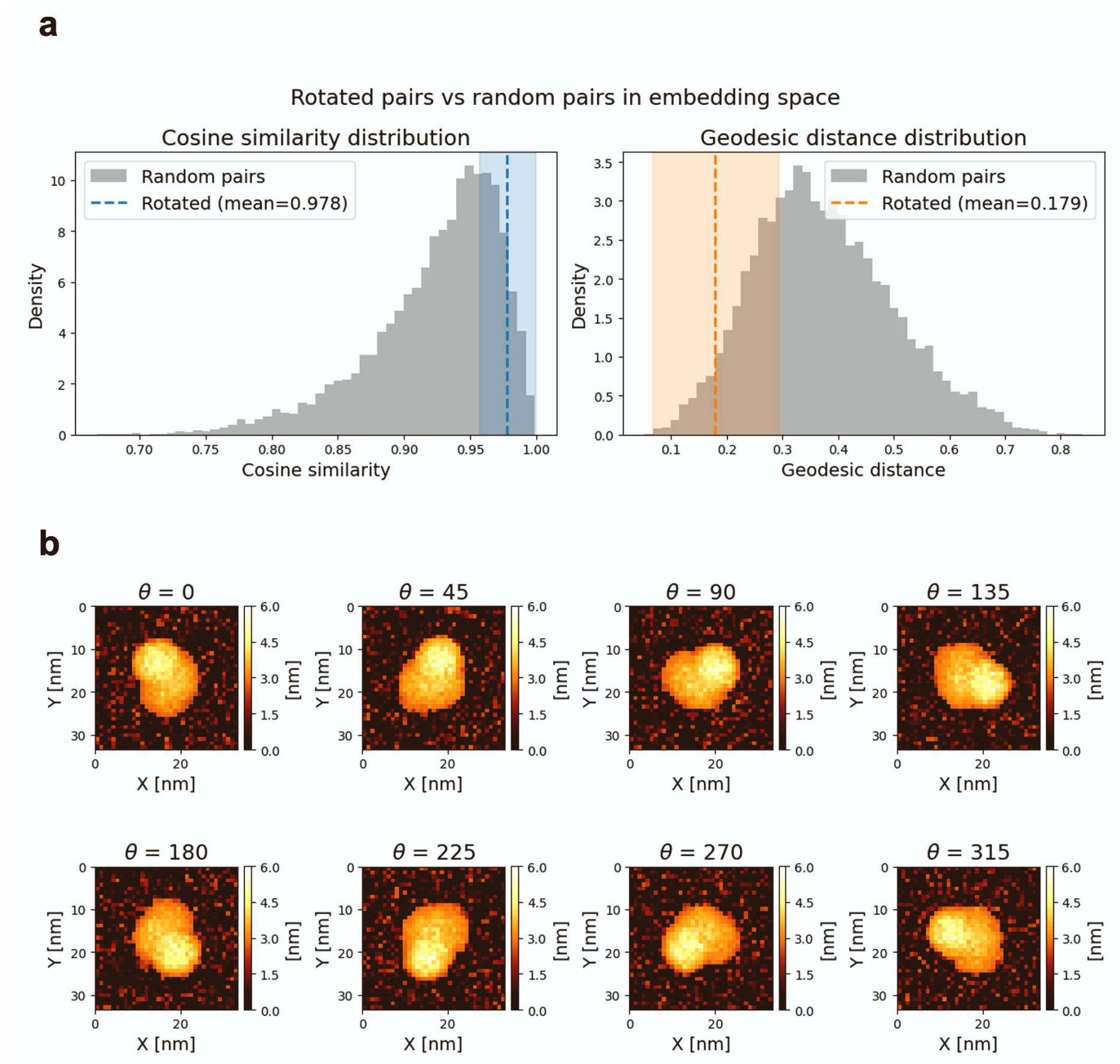

Figure S4: **Empirical rotation invariance of ResNet-18 embeddings**. For each molecular pose, simulated AFM images were generated at 8 in-plane rotation angles, and pairwise cosine similarity and geodesic distance in the embedding space were computed. Rotation pairs: cosine similarity 0.978 ± 0.021, geodesic distance 0.179 ± 0.113. Random (baseline) pairs: cosine similarity 0.924 ± 0.049, geodesic distance 0.374 ± 0.125. The substantially higher cosine similarity and lower geodesic distance for rotation pairs, well separated from the random baseline, confirm that the model has acquired empirical rotation invariance despite having no built-in rotational symmetry.

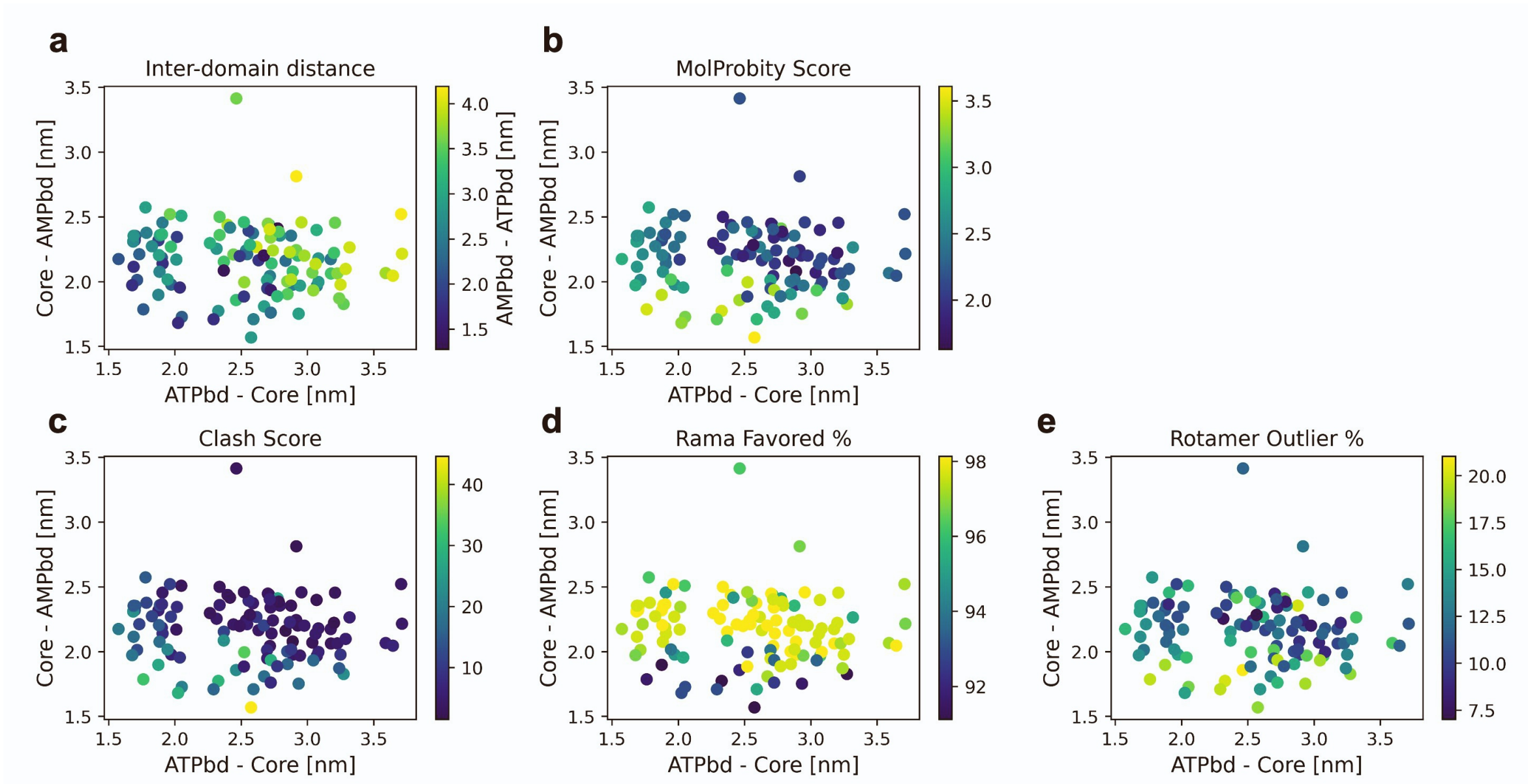

Figure S5: **Selected candidate conformations for AK and MolProbity evaluation for them. (a)** Projection of the candidate conformations into the inter-domain distance space. The following panels shows **(b)** MolProbity score, **(c)** Clash score, **(d)** Ramachandran favored rate (%), and **(e)** Rotamer outlier rate (%) of the candidate conformations.

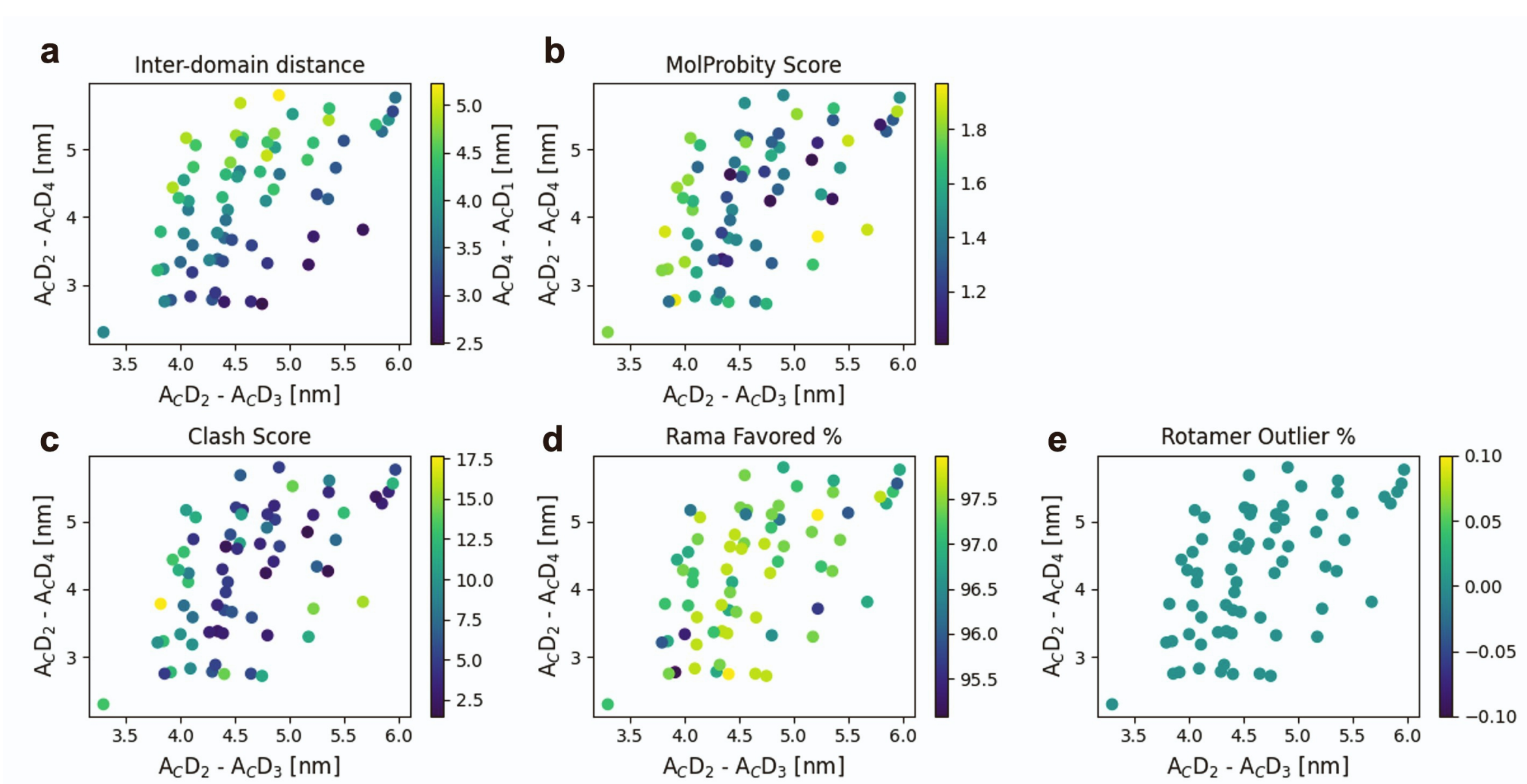

Figure S6: **Selected candidate conformations for FlhA$_C$ and MolProbity evaluation for them. (a)** Projection of the candidate conformations into the inter-domain distance space. The following panels shows **(b)** MolProbity score, **(c)** Clash score, **(d)** Ramachandran favored rate (%), and **(e)** Rotamer outlier rate (%) of the candidate conformations.

References

1. Advaith Maddipatla, Nadav Bojan Sellam, Meital Bojan, Sanketh Vedula, Paul Schanda, Ailie Marx and Alex M. Bronstein. "Inverse problems with experiment-guided AlphaFold". In: *arXiv* (2025). (preprint posted Feb 13, 2025). url: https://arxiv.org/abs/2502.09372.

2. Rishwanth Raghu, Axel Levy, Gordon Wetzstein and Ellen D. Zhong. “Multiscale guidance of AlphaFold3 with heterogeneous cryo-EM data”. In: *arXiv* (2025). (preprint posted Jun 4, 2025). url: https://arxiv.org/abs/2506.04490.
